# LncRNA MALAT1 promotes growth and metastasis of head and neck squamous cell carcinoma by repressing VHL through a non-canonical function of EZH2

**DOI:** 10.1038/s41419-023-05667-6

**Published:** 2023-02-22

**Authors:** Yuansheng Duan, Kai Yue, Beibei Ye, Peng Chen, Jin Zhang, Qinghua He, Yue Wu, Qingchuan Lai, Hong Li, Yansheng Wu, Chao Jing, Xudong Wang

**Affiliations:** 1 Department of Maxillofacial and Otorhinolaryngological Oncology, Tianjin Medical University Cancer Institute and Hospital, National Clinical Research Center for Cancer, Key Laboratory of Cancer Prevention and Therapy, Tianjin, Tianjin’s Clinical Research Center for Cancer, Tianjin, 300060 China; 2 Department of Bone and Soft Tissue Tumors, Tianjin Medical University Cancer Institute and Hospital, Tianjin, 300060 China

**Keywords:** Head and neck cancer, Metastasis, Prognostic markers

## Abstract

Long non-coding RNAs (LncRNAs) are implicated in malignant progression of human cancers. Metastasis-associated lung adenocarcinoma transcript 1 (MALAT1), a well-known lncRNA, has been reported to play crucial roles in multiple malignancies including head and neck squamous cell carcinoma (HNSCC). However, the underlying mechanisms of MALAT1 in HNSCC progression remain to be further investigated. Here, we elucidated that compared with normal squamous epithelium, MALAT1 was notably upregulated in HNSCC tissues, especially in which was poorly differentiated or with lymph nodes metastasis. Moreover, elevated MALAT1 predicted unfavorable prognosis of HNSCC patients. The results of in vitro and in vivo assays showed that targeting MALAT1 could significantly weaken the capacities of proliferation and metastasis in HNSCC. Mechanistically, MALAT1 inhibited von Hippel–Lindau tumor suppressor (VHL) by activating EZH2/STAT3/Akt axis, then promoted the stabilization and activation of β-catenin and NF-κB which could play crucial roles in HNSCC growth and metastasis. In conclusion, our findings reveal a novel mechanism for malignant progression of HNSCC and suggest that MALAT1 might be a promising therapeutic target for HNSCC treatment.

## Introduction

Head and neck cancer ranks the seventh common malignant tumor worldwide, and more than 90% of new cases are diagnosed as head and neck squamous cell carcinoma (HNSCC) [1], whose 5-year overall survival rate is only about 50% [2]. The unfavorable prognosis of patients suffered with HNSCC is attributed to severe local invasion and lymph node metastasis, in which multiple molecules are implicated. Therefore, a comprehensive understanding of the underlying mechanism of HNSCC metastasis is crucial for improving the clinical diagnosis and treatment, and even the prognosis of HNSCC patients.

Non-coding RNAs (ncRNAs), a class of transcripts that are coded by more than 75% of the human genome [3], mostly lack protein coding potential but play a critical role in physiological activities and diseases including cancer [4]. As one type of ncRNAs that are >200 nt long, long non-coding RNAs (LncRNAs) are well recognized to exhibit oncogenic and tumor-suppressive functions in various cancers [5]. Importantly, lncRNAs could be served as novel promising biomarkers and therapeutic targets for HNSCC and other tumors [6]. Metastasis associated lung adenocarcinoma transcript 1 (MALAT1) is a well-known lncRNA and was originally discovered in non-small cell lung cancer [7]. Previous studies have shown that MALAT1 is aberrantly expressed in numerous cancers and contributes to tumor malignant progression. In glioblastoma, MALAT1 acts as a competing endogenous RNA (ceRNA) for miR-199a to weaken the inhibitory effect of miR-199a on ZHX1, which strengthens tumor proliferation and progression [8], while MALAT1 also promotes autophagy-mediated chemoresistance by regulating miR-23b-3p/ATG12 axis in gastric cancer [9]. Moreover, YAP1-induced MALAT1 facilitates epithelial-mesenchymal transition (EMT) and angiogenesis by sponging miR-126-5p in colorectal cancer [10]. For HNSCC, many studies have revealed that MALAT1 could facilitate tumor progression through multiple mechanisms, mainly elevating oncogenic genes expression by serving as a ceRNA to sponge microRNAs [11]. Meanwhile, the effect of MALAT1 on tumor suppressors in HNSCC should also be explored.

In this study, we demonstrated that MALAT1 was markedly upregulated in HNSCC and correlated with lymph node metastasis and poor prognosis. Moreover, MALAT1 promoted HNSCC cell proliferation, invasion and metastasis in vitro and in vivo. MALAT1 regulated EZH2/STAT3/Akt axis to repress E3 ligase VHL, leading to the activation of β-catenin and NF-κB pathway. In general, these findings reveal that MALAT1 functions as an oncogene and potentially represents a promising therapeutic target in HNSCC.

## Materials and methods

### Human HNSCC specimens

A total of 157 cases of HNSCC surgical tissues were collected in Tianjin Medical University Cancer Hospital from 2009 to 2016. Tumor-node-metastasis (TNM) staging was determined according to the guideline of the American Joint Committee on Cancer International Union. This study was approved by the Ethics Committee of Tianjin Medical University Cancer Institute and Hospital, and informed consent was routinely obtained from the patients.

### Cell culture and reagents

HNSCC cell lines SCC15, SCC25 and CAL27 were purchased from American Type Culture Collection (ATCC, Manassas, VA, USA) and the TSCCA cell line was obtained from the Institute of Basic Medical Sciences, Chinese Academy of Medical Sciences. The UM1 (UM-SCC1) cell line were a gift from Professor Jinsong Hou (Sun Yat-sen University, Guangzhou, China). All cell lines were maintained in DMEM/Ham’s F12 (1:1), DMEM or RPMI 1640 supplemented with 10% FBS and penicillin (100 U/ml)/streptomycin (100 mg/ml) at 37 °C in a 5% CO_2_-humidified incubator. These cell lines above were authenticated by short tandem repeat (STR) genotyping and checked for *Mycoplasma* contamination before experiments. The small-molecule inhibitors targeting STAT3 (Stattic) and Akt (MK2206) were purchased from Selleck (Shanghai, China).

### Bioinformatics analysis

The levels of MALAT1 and VHL in cancerous tissues and adjacent normal counterparts were analyzed by using GEPIA (gepia.cancer-pku.cn) and GEO2R according to TCGA (The Cancer Genome Atlas) HNSCC database and GEO (Gene Expression Omnibus) datasets GSE83519 and GSE39366, respectively.

### Transfection

The siRNAs targeting MALAT1, EZH2 or VHL were purchased from RiboBio (Guangzhou, China), and the siRNAs sequences were shown in Supplementary Table S1. pCDH-hMALAT1 was purchased from Addgene (Cambridge, MA, USA), and pLVX-IRES-EZH2 was obtained from Tsingke (Beijing, China). Lipofectamine 3000 (Invitrogen) was used to deliver a pool of three siRNAs or plasmids into HNSCC cells according to the manufacturer’ s instructions. The cells were maintained with Opti-MEM (Gibco) without FBS during transfection, and then the medium was replaced with complete DMEM/F12 after 6 h.

### Cell growth assay and clonogenicity assay

For cell growth detection, the transfected SCC15 and UM1 cells (1500 cells per well) were seeded in 96-well plates. Then, cell number was counted at the indicated time (0, 24, 48, and 72 h). For colony formation assay, 2000 HNSCC cells were maintained with complete medium in a 10 cm plate for 2 weeks. The cells were washed, fixed, and then stained with 0.1% crystal violet. The colonies whose cell number was more than 50 were counted using an inverted microscope.

### Cell migration and invasion assay

For migration assay, 5 × 10^4^ HNSCC cells were plated into 24-well transwell chambers (Corning, NY, USA). For invasion assay, the upper chamber was pre-coated with Matrigel and 8 × 10^4^ cells were plated into transwell chambers. In both assays, the cells were maintained with 100 μl of medium without FBS in the upper chamber, and 600 μl of medium supplemented with 20% FBS was added into the lower chamber. After 24 h, the penetrated cells were fixed, stained, and counted in three random fields by using an inverted microscope (Leica Microsystems, Wetzlar, Germany).

### Wound healing assay

SCC15 and UM1 cells transfected with siRNAs were added in six-well plates and cultured to approximately 100% confluence. A 10 μl pipette tip was used to make scratches, and the cells were incubated with fresh medium without FBS for another 24 h. The wound closure was monitored at 0 and 24 h after scraping.

### Immunoblotting assay

Briefly, HNSCC cells were collected in RIPA lysis buffer containing protease and phosphatase inhibitors. 15–30 μg of protein was separated and transferred onto PVDF membranes, then blocked and probed using primary antibodies at 4 °C overnight. The antibodies against β-Catenin (1:1000), p-β-Catenin (Ser675) (1:1000), EZH2 (1:1000), P65 (1:1000), p-P65 (Ser536) (1:1000), p-STAT3 (1:1000), p-Akt (1:1000), H3K27me3 (1:1000), E-cadherin (1:1000), N-cadherin (1:1000), Vimentin (1:1000), Snail (1:1000), cleaved PARP (1:1000), cleaved Caspase-3 (1:1000), Cyclin D1 (1:1000), Histone3 (1:2000) were purchased from Cell Signaling Technology (Danvers, MA, USA). The antibodies against VHL (1:1000), GAPDH (1:5000) were obtained from Santa Cruz (Dallas, Texas, USA). At least three biologically independent experiments were performed.

### Immunofluorescence assay

After transfection, SCC15 and UM1 cells were seeded on cover glasses and cultured until they adhered to the surface. The cells were fixed with 4% paraformaldehyde and permeabilized with Triton X-100, then blocked with 5% BSA and incubated with primary antibodies overnight at 4 °C. Proteins were visualized by incubation with IgG conjugated to Alexa Fluor 488 or 594 (Cell Signaling Technology), and nuclei were stained with DAPI (Thermo Scientific) for 10 min at room temperature. All images were captured by using LSM 880 laser scanning confocal microscope (Zeiss, Oberkochen, Germany).

### Flow cytometry

For cell cycle detection, transfected HNSCC cells were fixed with 75% ethanol and washed twice with PBS, then incubated with propidium iodide (PI, 20 μg/ml) and RNase (100 μg/ml) at 37 °C in the dark for 30 min. For determining apoptosis rate, cells were trypsinized and resuspended as a single-cell suspension, then stained with FITC-Annexin V and PI according to the manufacturer’s instructions (BD Biosciences, Franklin Lakes, NJ, USA). All assays were measured on the same FACS Canto II (BD Biosciences).

### Quantitative real-time PCR (qPCR)

Total RNA was extracted from cells using TRIzol reagent (Invitrogen, Waltham, MA, USA) according to standard instructions. PrimeScript™ RT Master Mix (TaKaRa, Shiga, Japan) was used to transcribe RNA to cDNA. Quantitative Real-time PCR (qPCR) was performed using SYBR Premix Ex TaqTM II (TaKaRa) on 7500 Real-Time PCR system (Applied Biosystems, Foster City, CA, USA). GAPDH was served as an internal control for normalization and the 2^−ΔΔCt^ method was used to calculate gene expression. The following primers were used in qPCR assay:

5′-AGGGCTGCTTTTAACTCTG-3′ (GAPDH, Forward),

5′-CTGGAAGATGGTGATGGG-3′ (GAPDH, Reverse),

5′-GACGGAGGTTGAGATGAAGC-3′ (MALAT1, Forward),

5′-ATTCGGGGCTCTGTAGTCCT-3′ (MALAT1, Reverse),

5′-ACATCGTCAGGTCGCTCTAC-3′ (VHL, Forward),

5′-ATCTCCCATCCGTTGATGTG-3′ (VHL, Reverse).

### Chromatin immunoprecipitation sequencing (ChIP-seq) and ChIP-qPCR

SCC15 cells transfected with si-MALAT1 or negative control were collected to conduct a ChIP assay using SimpleChIP® Plus Enzymatic Chromatin IP Kit (9005, Cell Signaling Technology) with anti-H3K27me3 antibody (Cell Signaling Technology, ChIP grade) according to the manufacturer’s protocol. ChIP-seq libraries were generated using NEBNext® Ultra II DNA Library Prep Kit for Illumina (New England Biolabs, MA, USA) and sequenced on Illumina NovaSeq 6000 (Mingma Technologies, Shanghai, China).

ChIP assays were conducted by using EZ-Magna ChIP Kit (17-10086, Merck, Darmstadt, Germany) with anti-H3K27me3 antibody. The qPCR assay was applied to analyze the enriched DNA.

### RNA in situ hybridization (RNA-ISH)

RNA-ISH experiment was assayed using a RNAscope® Probe-Hs-MALAT1 kit (#400811, Advanced Cell Diagnostics, Hayward, CA) according to the manufacturer’s instructions.

### Immunohistochemistry staining

For immunohistochemistry (IHC) staining, paraffin-embedded HNSCC tissue samples were deparaffinized and rehydrated. Endogenous peroxidase was blocked by 3% H_2_O_2_, then citrate-based antigen retrieval was conducted. The samples were incubated with primary antibodies against VHL, EZH2, p-STAT3 and p-Akt overnight at 4 °C. After incubation with HRP-conjugated secondary antibody, the sections were stained using DAB substrate kit according to the manufacture’s instruction.

### TUNEL assay

TUNEL assay was performed to detect the apoptotic ratio of HNSCC cells by using a cell death kit (Beyotime, Shanghai, China). Cells were visualized using an Imager.Z2 immunofluorescence microscope (Zeiss).

### Xenograft tumor model

Animal experimental protocols were approved by Animal Care and Use Committee of Tianjin Medical University Cancer Institute and Hospital. 5 × 10^5^ SCC15 cells were injected into the instep of the 5-week-old BALB/c male nude mice. Until xenograft establishment, mice were double-blindly, randomly divided into two groups (7 mice per group) and treated with the negative control oligos or in vivo siRNAs against MALAT1 (5 nmol/20 g, RiboBio, Guangzhou, China) every 4 days through intratumor injection, respectively. The tumor volume was measured using a formula (volume = (long diameter × short diameter^2^)/2). After 50 days. the mice were sacrificed, xenograft tumors and inguinal lymph node were collected for formalin fixation and paraffin-embedding.

### Statistical analysis

All assays except IHC and animal experiments were repeated three times at least. The statistical analyses were performed using GraphPad Prism 6. Data were presented as mean ± standard deviation (SD) and analyzed by using two-tailed *Student’s t*-test unless stated particularly when the data met the assumptions of the tests. The difference for which *P* < 0.05 was considered statistically significant.

## Results

### Elevated LncRNA MALAT1 indicates unfavorable prognosis of HNSCC patients

**Fig. 1:**
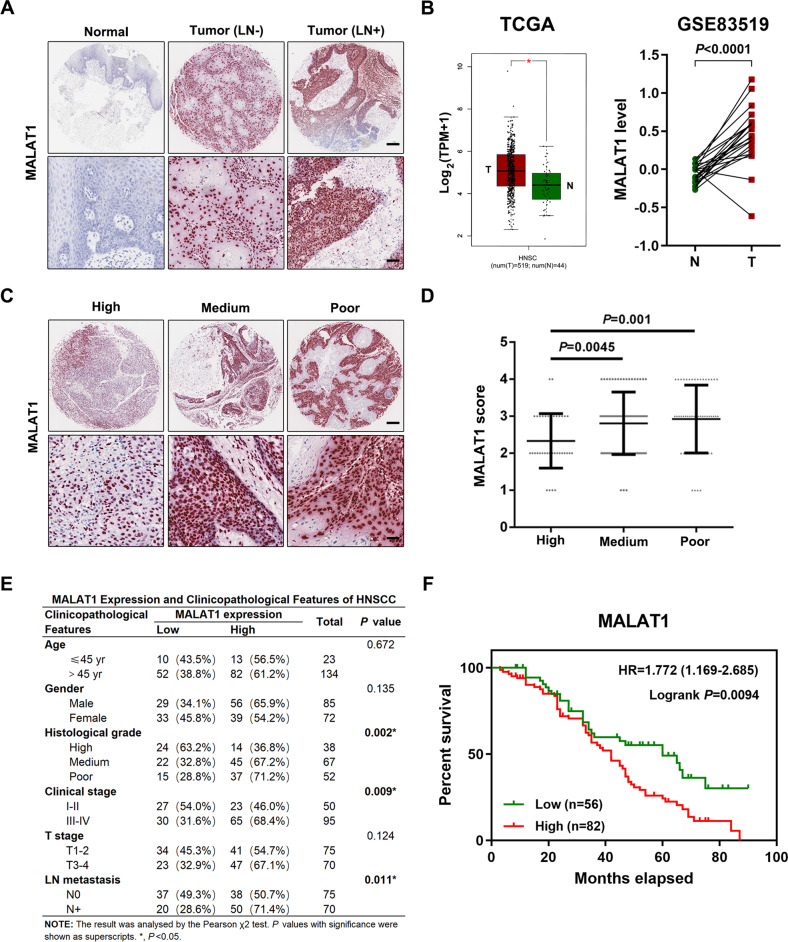
MALAT1 is a potential biomarker for HNSCC progression and prognosis. **A** Representative ISH images of MALAT1 in HNSCC specimens (with/without lymph node metastasis) and adjacent normal tissues. Scale bar, 250 μm (upper) and 25 μm (lower). LN−, without lymph node metastasis. LN+, with lymph node metastasis. **B** The bioinformatic analysis of TCGA and GEO (GSE83519) databases showed that compared with normal tissues, MALAT1 was upregulated in HNSCC tissues. Data, mean ± SD, *P < 0.05. T tumor tissues. N normal tissues. TPM transcripts per million. **C**, **D** The results of ISH staining showed that the level of MALAT1 correlated negatively with the differentiation degree of HNSCC. Scale bar, 250 μm (upper) and 25 μm (lower). Data, mean ± SD. High, high differentiated. Medium, medium differentiated. Poor, poor differentiated. **E** The correlation between MALAT1 expression and clinicopathological features of HNSCC. 157 cases of HNSCC specimens were analyzed. The results were analyzed by the Pearson χ^2^ test. *P < 0.05. **F** Kaplan–Meier survival curve suggested that enhanced MALAT1 indicated poor prognosis of HNSCC patients in our cohort. HR hazard rate.

Firstly, the expression of MALAT1 was evaluated in HNSCC tissues. As shown in Fig. 1A, MALAT1 was enriched in the nucleus of HNSCC cells as observed by RNA-ISH. Compared with adjacent normal tissues, MALAT1 was aberrantly expressed in HNSCC specimens (Fig. 1A and Supplementary Fig. 1). In addition, the positive staining of MALAT1 in HNSCC samples with lymph node metastasis was much stronger than that in the ones without lymph node metastasis (Fig. 1A and Supplementary Fig. 2). Meanwhile, we also analyzed the abundance of MALAT1 based on TCGA-HNSCC database and one GEO dataset concerning the expression profiles in HNSCC tissues (GSE83519). Then, we found that the expression of MALAT1 significantly elevated in HNSCC samples (Fig. 1B), which was in line with the results of our cohort stated above. Moreover, an obvious increase of MALAT1 was observed in patients with moderately/poorly differentiated HNSCC (Fig. 1C, D). Further analysis revealed that MALAT1 expression significantly correlated with histological grade, clinical stage and lymph node metastasis in HNSCC (Fig. 1E), suggesting that MALAT1 played a crucial role in the malignant progression of HNSCC. Kaplan-Meier curve showed that HNSCC patients with MALAT1 overexpression had a shorter overall survival (Fig. 1F, HR = 1.769, *P* = 0.0137). As shown in Supplementary Table S2, the overall survival (OS) of HNSCC patients was significantly relative to histological grade, clinical stage, T stage, LN metastasis besides MALAT1 expression. Then, multivariate Cox regression analysis showed that histological grade, LN metastasis and MALAT1 expression could be considered as independent risk factors for OS of HNSCC patients (Supplementary Table S3). Collectively, these results indicate that MALAT1 is overexpressed in HNSCC and predicts a poor outcome.

### Depletion of MALAT1 inhibits proliferation and promotes apoptosis in HNSCC in vitro

**Fig. 2:**
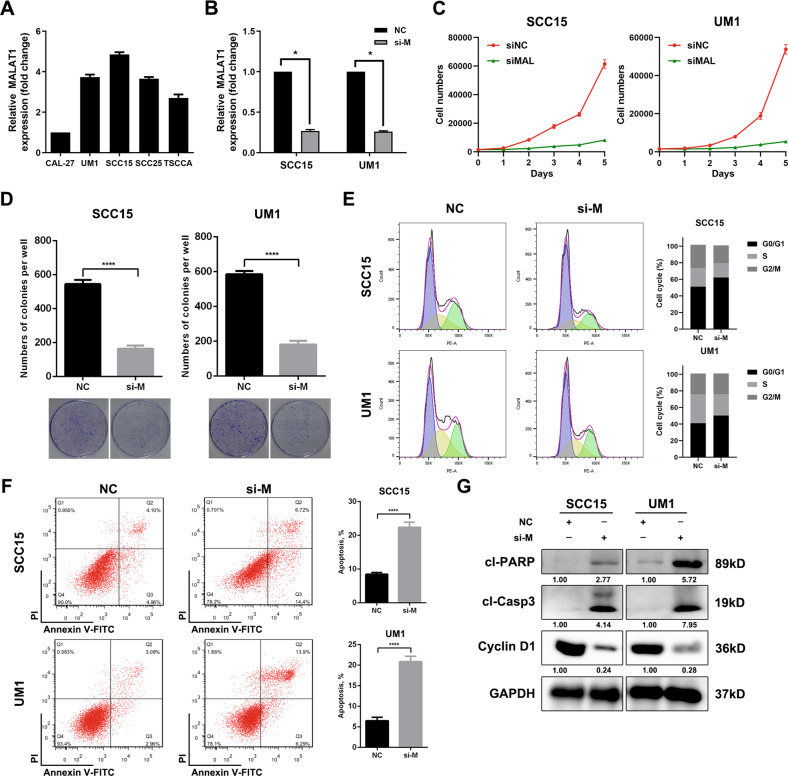
MALAT1 depletion inhibits proliferation and promotes apoptosis of HNSCC cells in vitro. **A** The level of MALAT1 was measured in a panel of HNSCC cell lines using qPCR assay. **B** The expression of MALAT1 was detected using qPCR assay in the SCC15 and UM1 cells transfected with a pool of three siRNAs targeting MALAT1. **C** The effect of MALAT1 knockdown on the growth of SCC15 and UM1 cells in vitro was assessed through cell counting. Bar, SD for quintuplicate wells. siNC, negative control for siRNA. siMAL, si-MALAT1. **D** MALAT1 depletion significantly weakened the capacity of colony formation of HNSCC cells. Bar, SD for triplicate wells. **E** The results of flow cytometry showed that MALAT1 silencing induced G1 phase arrest in SCC15 and UM1 cells. **F** HNSCC cells were transfected with MALAT1 siRNAs for 48 h, then cell apoptosis rate was measured by flow cytometry. **G** The abundance of cleaved PARP, cleaved Caspase-3 and Cyclin D1 was probed in MALAT1-depleted HNSCC cells and the negative control group. Data in this figure, mean ± SD, *P < 0.05, **P < 0.01, ****P < 0.0001. NC negative control. si-M, si-MALAT1.

Next, we further explored the biological functions of MALAT1 in HNSCC. As shown in Fig. 2A, MALAT1 was highly expressed in a panel of HNSCC cell lines, especially in SCC15 and UM1 cell lines. Then, we delivered a pool of three specific siRNAs into these two cells for targeting MALAT1 (Fig. 2B). The results showed that MALAT1 depletion significantly inhibited the proliferation of SCC15 and UM1 cells (Fig. 2C). Besides, the number of colonies in vitro was strikingly reduced in the MALAT1-silenced HNSCC cells compared with negative control (Fig. 2D), while MALAT1 overexpression strengthened the capacity of colony formation of CAL27 cells (Supplementary Fig. 3A, B). Through cell-cycle distribution detection, we observed an increase in the accumulation of cells in the G1 phase in SCC15 and UM1 cells transfected with siRNAs targeting MALAT1 (Fig. 2E), indicating that MALAT1 silencing could induce G1 phase arrest of HNSCC cells. Additionally, the effect of MALAT1 on HNSCC cell apoptosis was determined by using flow cytometry. As shown, the apoptosis rate was dramatically increased in the si-MALAT1-transfected cells (Fig. 2F). We also assessed the levels of cell cycle-related molecule Cyclin D1 and apoptosis-related protein Caspase-3 using western blot assay. After the decrease of MALAT1, the expression of Cyclin D1 was reduced, while the abundance of cleaved PARP and cleaved Caspase-3 rose remarkably (Fig. 2G and Supplementary Fig. 4). These data show that MALAT1 drives cell proliferation and suppress apoptosis of HNSCC cells.

### MALAT1 silencing impairs the capacities of migration and invasion of HNSCC cells

**Fig. 3:**
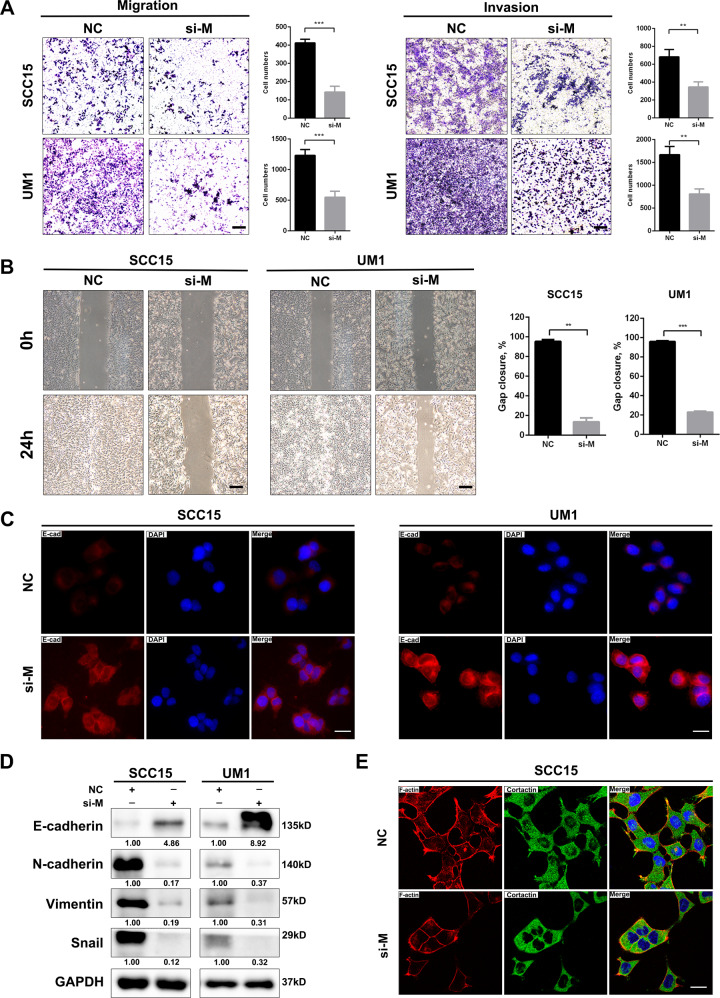
MALAT1 silencing weakens the motility capacity of HNSCC cells in vitro. **A** Transwell assay showed that MALAT1 knockdown hindered the migration and invasion of SCC15 and UM1 cells. Scale bar, 100 μm. **B** Representative images of gaps in HNSCC cells transfected with si-MALAT1 and negative control at the beginning (0 h) and the endpoint (24 h) of wound healing assay were shown. MALAT1 depletion retarded the wound-healing process in HNSCC cells. Scale bar, 200 μm. **C** The immunofluorescence staining of E-cadherin in SCC15 and UM1 cells transfected with MALAT1 siRNAs and negative control, respectively. Scale bar, 20 μm. **D** The proteins of E-cadherin, N-cadherin, Vimentin and Snail were determined by western blot assay in si-MALAT1-transfected HNSCC cells. **E** Representative images of immunofluorescence of F-actin and Cortactin in SCC15 cells transfected with si-NC or si-MALAT1 were shown. Scale bar, 20 μm. Data in this figure, mean ± SD, **P < 0.01, ***P < 0.001. NC negative control. si-M si-MALAT1.

Metastasis is a major contributor to cancer-caused death in patients suffered with HNSCC. Therefore, we further performed a series of experiments in vitro to evaluate the effect of MALAT1 on migration and invasion of HNSCC cells. Upon delivery of si-MALAT1 into SCC15 and UM1 cells, the migration and invasion of these recipient cells were dramatically hindered relative to negative control cells (Fig. 3A). Besides, MALAT1 depletion retarded the healing velocity of HNSCC cells within 24 h (Fig. 3B). Immunofluorescence staining showed that E-cadherin, an epithelial marker, was obviously increased in the membrane of MALAT1-silenced SCC15 and UM1 cells (Fig. 3C), which was reconfirmed by immunoblotting shown in Fig. 3D. In addition to E-cadherin, the protein levels of mesenchymal markers were also examined with the knockdown of MALAT1. We observed that targeting MALAT1 resulted in the downregulation of N-cadherin, Vimentin and Snail (Fig. 3D), suggesting that MALAT1 depletion could reverse epithelial-mesenchymal transition (EMT) process. Moreover, si-MALAT1 also inhibited the formation of invadopodia in the HNSCC cells (Fig. 3E and Supplementary Fig. 5, white arrows). On the contrary, MALAT1 overexpression promoted migration, invasion and EMT in CAL27 cells (Supplementary Fig. 3C, D). Together, these findings demonstrate that MALAT1 could strengthen the motility ability of HNSCC cells in vitro.

### Blockade of MALAT1 inactivates β-catenin and NF-κB pathways by upregulating VHL

The comprehensive genomic landscape of HNSCC provided by TCGA have shed light on the crucial roles of both β-catenin and NF-κB signaling in HNSCC progression [12]. In these two pathways, activated β-catenin and P65 both translocate into the nucleus to regulate gene transcription. Therefore, we detected the effect of MALAT1 on the activation of β-catenin and P65. In MALAT1-depleted HNSCC cells, the expressions of β-catenin, p-β-catenin, P65 and p-P65 were strikingly reduced (Supplementary Fig. 6A). Through western blot of cytosolic/nuclear fractionation and immunofluorescence assay, we found that silencing of MALAT1 dramatically suppressed the nuclear translocation and activation of β-catenin and P65, respectively (Supplementary Fig. 6B, C).

**Fig. 4:**
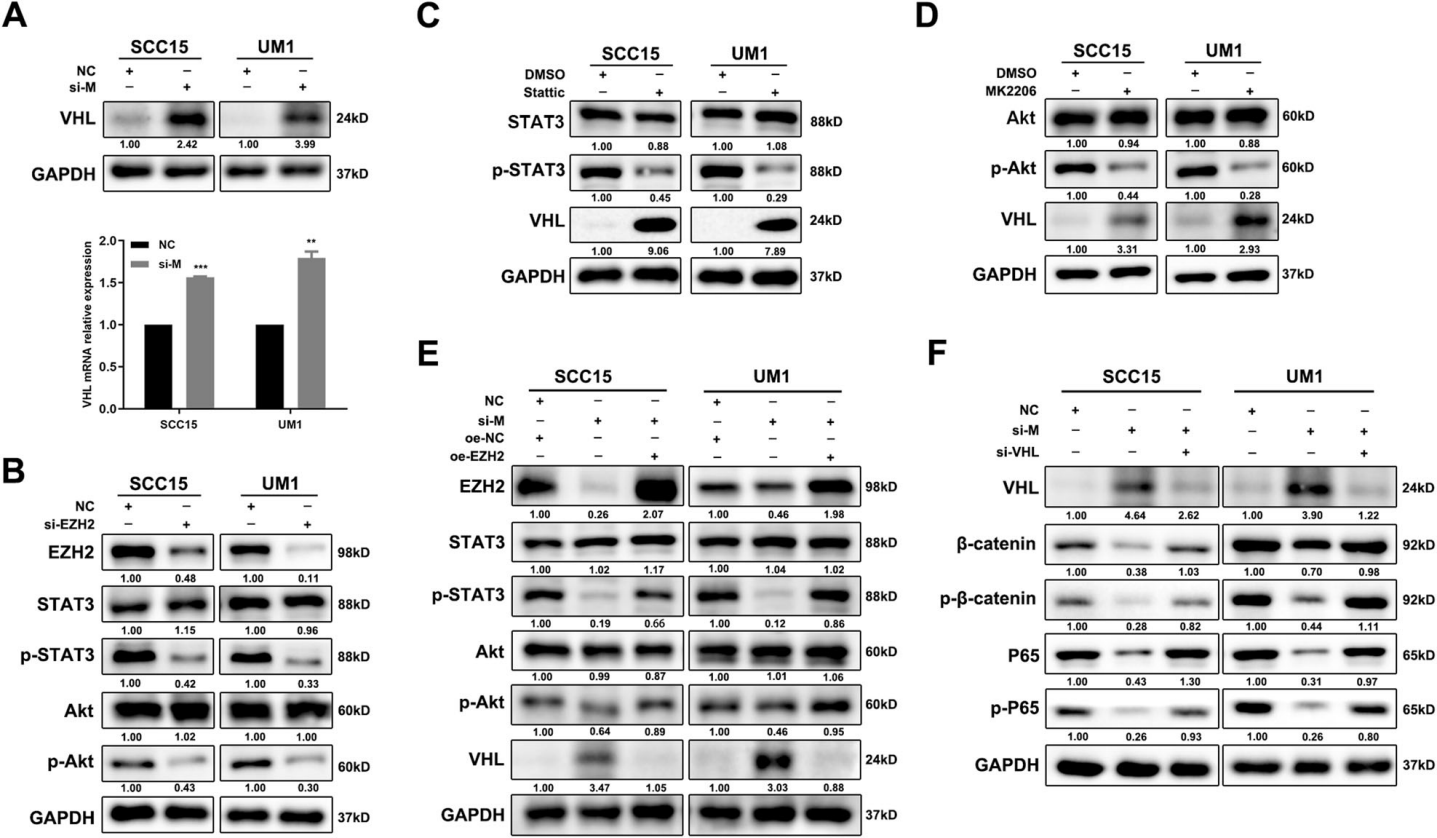
MALAT1 represses VHL to activate β-catenin and NF-κB pathways through a non-canonical function of EZH2. **A** The protein and mRNA levels of VHL were detected in MALAT1-depleted HNSCC cells by using western blot and qPCR assays. Data, mean ± SD, **P < 0.01, ***P < 0.001. **B** The abundance of EZH2, p-STAT3 and p-Akt was measured in SCC15 and UM1 cells transiently transfected with si-EZH2 and negative control, respectively. The protein expression of VHL was elevated using immunoblotting assay in HNSCC cells treated with stattic (**C**) and MK2206 (**D**). **E** The results of western blot revealed that EZH2 overexpression rescued the effect of si-MALAT1 on p-STAT3, p-Akt, and VHL, respectively. oe overexpression. **F** The results of western blot showed that si-VHL mitigated the inhibition of si-MALAT1 on β-catenin and NF-κB pathways. NC negative control. si-M si-MALAT1.

VHL, as a E3 ligase, has been reported to induce the protein degradation of β-catenin and P65 to inhibit tumor progression. Hence, we speculated that MALAT1 might elevate β-catenin and P65 protein expression by repressing VHL. To support our hypothesis, we transfected specific siRNAs targeting MALAT1 into SCC15 and UM1 cells, and found that the protein and mRNA levels of VHL were both obviously increased (Fig. 4A). Li et al. has verified that nuclear MALAT1 could reinforce trimethylation modification on histone H3 lysine27 (H3K27me3) to suppress gene transcription in a EZH2-dependent manner [13]. As expected, the levels of EZH2 and H3K27me3 were both decreased in the MALAT1-silenced HNSCC cells (Supplementary Fig. 7A). We also observed that si-EZH2, resembling the function of si-MALAT1, led to the downregulation of H3K27me3 and the upregulation of VHL (Supplementary Fig. 7B). Subsequently, we performed ChIP-seq to generate genome-wide maps of H3K27me3 enrichment in SCC15 cells. However, no obvious H3K27me3 peak across the body of *VHL* gene was observed in MALAT1-silenced SCC15 cells or negative control (Supplementary Fig. 7C), which was further confirmed by ChIP assay. There was no enrichment of H3K27me3 in VHL promoter detected in SCC15 cells (Supplementary Fig. 7D). In addition to the role of histone methyltransferase, EZH2 also regulate the activation of critical signaling pathways, including STAT3 and Akt signaling, which was verified in our study (Fig. 4B). Then, small-molecule inhibitors (SMI) Stattic and MK2206 were used to inactivate STAT3 and Akt, respectively. A dramatical increase of VHL was measured in SMI-treated HNSCC cells (Fig. 4C, D). Once EZH2 was overexpressed, the influence of si-MALAT1 on p-STAT3, p-Akt, and VHL was rescued (Fig. 4E and Supplementary Fig. 8), implying that MALAT1 could attenuate VHL expression through EZH2/STAT3/Akt axis. Furthermore, the delivery of si-VHL could restore the expression and activation of β-catenin and P65 (Fig. 4F). Consistent with our hypothesis, MALAT1 promotes the activation of β-catenin and NF-κB pathways by suppressing VHL through a non-canonical function of EZH2 in HNSCC.

### VHL downregulation is essential for MALAT1-mediated malignancies of HNSCC cells

**Fig. 5:**
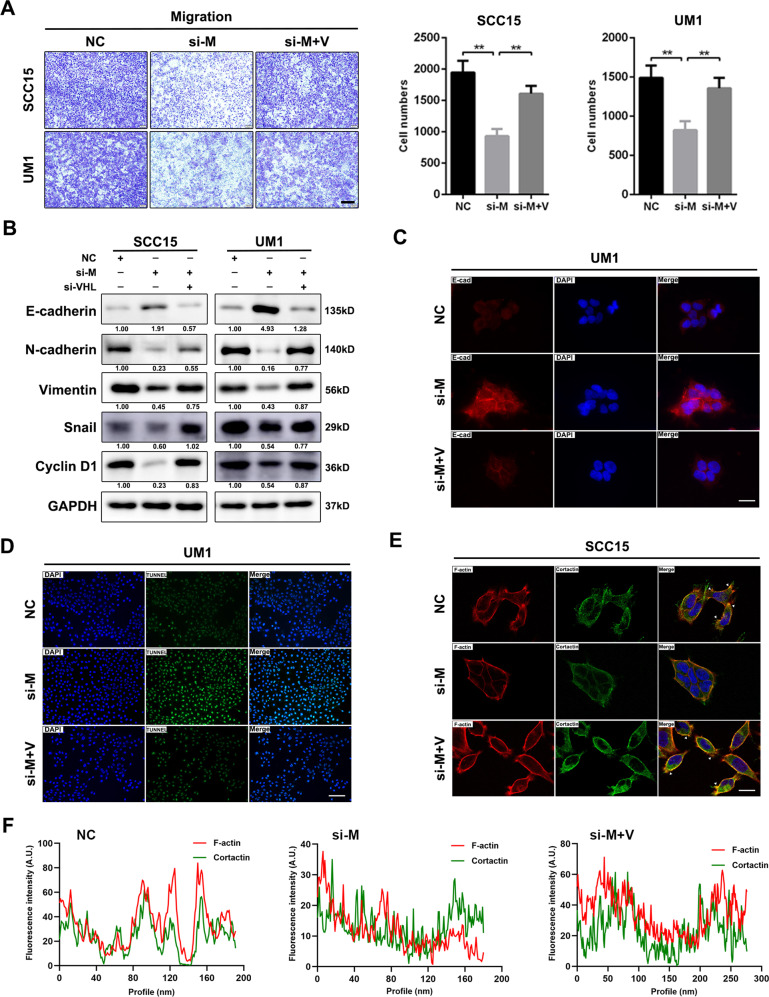
VHL downregulation is implicated in MALAT1-mediated HNSCC progression. **A** The transwell assay results showed that VHL silencing promoted the migration of si-MALAT1-transfected SCC15 and UM1 cells. The number of penetrated cells was counted. Scale bar, 100 μm. Data, mean ± SD, **P < 0.01. **B** Transfection with si-VHL was performed in si-MALAT1-transfected SCC15 and UM1 cells. Then, the abundance of E-cadherin, N-cadherin, Vimentin, Snail, and Cyclin D1 was measured in indicated groups. **C** The immunofluorescence staining of E-cadherin in UM1 cells transfected with si-MALAT1 or si-MALAT1/si-VHL was shown, respectively. Scale bar, 20 μm. **D** The results of TUNEL assay suggested that VHL knockdown inhibited si-MALAT1-induced apoptosis of UM1 cells. Scale bar, 100 μm. **E** The representative immunofluorescence images of F-actin and cortactin in SCC15 cell transfected with si-MALAT1, si-MALAT1/si-VHL and negative control, respectively. Scale bar, 20 μm. **F** The Fluorescence intensities of F-actin and cortactin in indicated groups as described in (**E**) were determined using ImageJ software. NC negative control. si-M si-MALAT1. si-V si-VHL.

As VHL was proved to be regulated by MALAT1 which dramatically promoted malignant progression of HNSCC, we then examined whether VHL was implicated in this regulation. We reduced VHL expression in both SCC15 and UM1 cells transfected with si-MALAT1, and observed that decreased level of VHL could cushion the inhibitory effect of si-MALAT1 on proliferation, migration and invasion (Supplementary Fig. 9, Fig. 5A and Supplementary Fig. 10). Besides, the expressions of N-cadherin, Vimentin, Snail and Cyclin D1 were restored by si-VHL transfection in MALAT1-silenced HNSCC cells (Fig. 5B and Supplementary Fig. 11); while, VHL knockdown led to a decrease of E-cadherin in si-MALAT1-transfected HNSCC cells (Fig. 5B, C and Supplementary Fig. 12). The results of TUNEL staining indicated that the number of apoptotic cells in HNSCC cells co-transfected with si-MALAT1 and si-VHL was obviously less than that in the ones transfected with si-MALAT1 only (Fig. 5D and Supplementary Fig. 13). As shown in Fig. 5E and Supplementary Fig. 14, VHL silencing strengthened the co-localization between F-actin and Cortactin along the cellular membrane in MALAT1-depleted HNSCC cells (white arrows), indicating the formation of invadopodia was improved. These data illustrate that MALAT1-mediated downregulation of VHL could promote HNSCC progression.

**Fig. 6:**
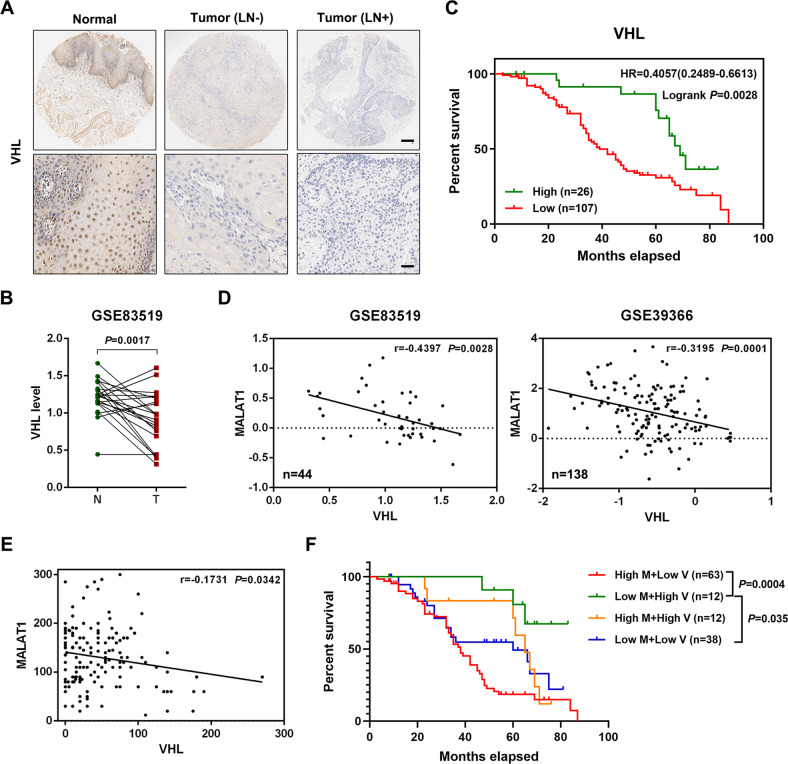
VHL is downregulated in HNSCC tissues and negatively correlates with MALAT1. **A** Representative IHC images of VHL in HNSCC specimens (with/without lymph node metastasis) and adjacent normal tissues. Scale bar, 250 μm (upper) and 25 μm (lower). LN−, without lymph node metastasis. LN+, with lymph node metastasis. **B** The bioinformatic analysis of GSE83519 databset showed that the level of VHL in normal tissues was significantly higher than that in HNSCC tissues. Data, mean ± SD. T tumor tissues. N normal tissues. **C** Kaplan–Meier plot determined that decreased VHL suggested unfavorable HNSCC outcome in our cohort (HR = 1.769, *P* = 0.0006). **D** The expression correlation between MALAT1 and VHL was analyzed based on the data of two GEO databases (GSE83519 and GSE39366). **E** The analysis of ISH and IHC staining indicated that MALAT1 expression negatively correlated with the level of VHL in our HNSCC cohort. **F** Detailed Kaplan–Meier plot was conducted according to the level of both MALAT1 and VHL in our HNSCC cohort. The results showed that high MALAT1 expression/low VHL expression predicted shorter overall survival of patients with HNSCC. High M high MALAT1 expression. Low M low MALAT1 expression. High V high VHL expression. Low V low VHL expression.

Then, we further detected the level of VHL in HNSCC tissues. The IHC staining showed that compared with normal tissues, VHL expression was obviously reduced in HNSCC specimens, especially in the ones with lymph node metastasis (Fig. 6A). The analysis of GSE83519 also indicated a lower level of VHL in HNSCC samples (Fig. 6B). Besides, the HNSCC patients with higher expression of VHL had a better overall survival (Fig. 6C), suggesting that VHL could serve as a favorable prognosis predictor. Meanwhile, the relationship between MALAT1 and VHL in HNSCC was assessed by analyzing public database and IHC results we obtained. As expected, MALAT1 negatively correlated with VHL in HNSCC according to the data of both GEO datasets (GSE83519 and GSE39366) and our cohort (Fig. 6D, E). Moreover, further stratification of HNSCC patient groups based on high MALAT1/low VHL expression improved the predictive capability of either one (Fig. 6F).

### Targeting MALAT1 suppresses tumor growth and lymph node metastasis in HNSCC

**Fig. 7:**
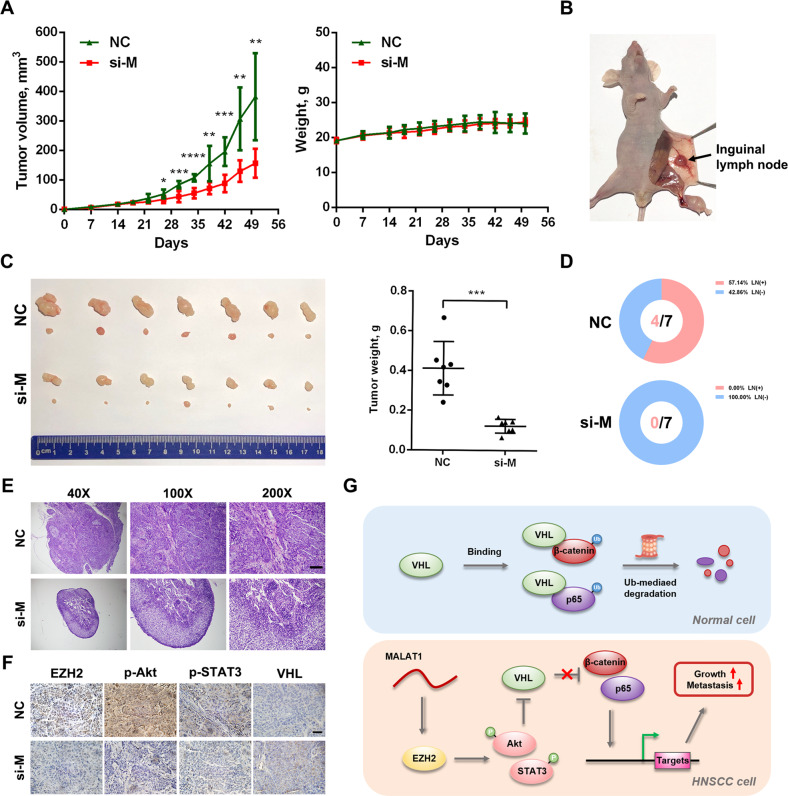
In vivo siRNAs targeting MALAT1 inhibits HNSCC tumor growth and lymph node metastasis. **A** Tumor volume and body weight were measured every 4 days to determine the effect of in vivo si-MALAT1 on xenografts growth and mice body weight. **B** A representative image for xenografts in instep and inguinal lymph node of mouse. **C** Representative images for indicated treated HNSCC xenografts (upper) and inguinal lymph nodes (lower). The size and weight of xenografts were significantly decreased after the treatment of in vivo si-MALAT1. **D**, **E** MALAT1 inhibition by in vivo siRNAs suppressed inguinal lymph node metastasis of HNSCC xenografts. The representative H&E images of inguinal lymph nodes in indicated groups were shown. Scale bar, 50 μm. **F** The IHC staining revealed that in vivo si-MALAT1 inhibited the levels of EZH2, p-Akt and p-STAT3, and promoted VHL expression. Scale bar, 25 μm. **G** Schematic of the mechanism of MALAT1/EZH2/VHL axis for promoting HNSCC growth and metastasis.

To clarify whether MALAT1 could be a therapeutic target for HNSCC in vivo, we evaluated the potential clinical efficacy of MALAT1 depletion using a mouse model of lymph node metastasis. Until the SCC15 cells were xenografted into the instep for 14 days, the mice were treated with negative control oligos or in vivo siRNAs targeting MALAT1. As shown in Fig. 7A, compared with the control group, MALAT1 siRNAs significantly inhibited the tumor growth of xenografts, while no difference was observed in the body weight between two groups. Then, we collected the xenografts and inguinal lymph nodes (Fig. 7B) and found that the tumor weight of xenografts treated with MALAT1 siRNAs was much lighter than that of negative control (Fig. 7C). Moreover, targeting MALAT1 strikingly impeded the lymph node metastasis of xenografts on the basis of H&E staining in inguinal lymph node (Fig. 7D, E). Subsequently, the in vivo siMALAT1 dramatically reduced the level of MALAT1 in xenografts (Supplementary Fig. 15), and IHC staining showed that si-MALAT1 obviously reduced EZH2, p-STAT3, p-Akt expression, and elevated VHL level in SCC15 xenografts (Fig. 7F). Taken together, these results suggest that MALAT1 could be served as a promising target to inhibit HNSCC growth and lymph node metastasis.

## Discussion

Previous studies have demonstrated that many lncRNAs display aberrant expression in various cancers and also possess strong promise toward the development of novel biomarkers and therapeutics. In colorectal cancer, MNX1-AS1, a novel colorectal cancer-related lncRNA, is significantly overexpressed and predicts unfavorable prognosis. Once MNX1-AS1 is inhibited, the in vivo growth of colorectal cancer is dramatically suppressed [14]. Wu et al. has confirmed that oncogenic lncRNA LEISA is highly expressed in, and correlates with clinical progression and poor outcome of lung adenocarcinoma (LAD). Through enhancing STAT3-induced expression of IL-6, LEISA could promote tumor progression [15], which reveals the crucial role of LEISA in LAD. For lncRNA MALAT1, its aberrant expression has been found in multiple cancers, including lung cancer [16], hepatocellular carcinoma [17], colorectal cancer [18], gastric cancer [19]. In this study, we found that MALAT1 was significantly upregulated in HNSCC through both ISH assay and public databases analysis. Moreover, MALAT1 was significantly associated with histological grade, clinical stage, T stage, lymph node metastasis and poor prognosis in HNSCC, suggesting that MALAT1 may be implicated in the malignant progression of HNSCC. Besides, the growth and lymph node metastasis, the major contributors to cancer-caused death in HNSCC, were dramatically inhibited by targeting MALAT1. Therefore, our work confirmed that MALAT1 could be served as a potential diagnostic biomarker and therapeutic target for HNSCC.

Comprehensive genomic characterization of HNSCC has identified various key pathways and components [12]. Among them, P65 and β-catenin could both exhibit oncogenic functions as transcriptional factors. For canonical NF-κB pathway, the IKK complex-mediated phosphorylation induces the degradation of IκB and abolishes its inhibition on P65, then P65 is activated and translocates into nucleus to regulate gene transcription [20]. In canonical Wnt/β-catenin signaling, the destruction complex which consists of Axin, APC, GSK3β, and CK1α mediates the proteasomal degradation of β-catenin; when Wnt ligands bind to Fzd receptors and LRP co-receptors, the function of destruction complex is impaired by Dvl protein polymers, resulting in the stabilization and accumulation of β-catenin which then translocates into the nucleus to form an active transcriptional complex with LEF and TCF proteins [21]. Our previous studies have illuminated the critical roles of P65 and β-catenin in head and neck cancer [22, 23], so we explored whether MALAT1 promoted HNSCC progression by regulating these two pathways. The results of cell fractionation and immunofluorescence staining showed that MALAT1 depletion dramatically reduced the expression and nuclear accumulation of P65 and β-catenin. In the meanwhile, we found the phosphorylation of P65 at Ser536 and β-catenin at Ser675, the key amino acid residues for the activation of these two pathways [24, 25], was inhibited by MALAT1 depletion, which further suggested that MALAT1 could activate P65 and β-catenin to promote HNSCC progression.

The Von Hippel–Lindau gene (VHL), first identified in 1993, is the causal gene for VHL disease [26]. Accumulating evidences have suggested that VHL acts as a tumor suppressor in many types of human cancers. Mutation, silencing, deletion or methylation of VHL contributes to tumorigenesis and cancer progression [27]. VHL serves as a substrate recognition component of an E3 ubiquitin ligase complex that targets HIFα for ubiquitination and degradation [28]. In addition to HIFα, VHL also inhibits NF-κB signaling by mediating K63-ubiquitination of IKKβ [29], and our previous study have verified that elevated VHL could inactivate β-catenin pathway, and impair the capacities of proliferation and invasion in oral squamous cell carcinoma [30]. In this article, MALAT1 was proven to inhibit VHL expression, which promoted the activation of P65 and β-catenin and HNSCC progression. Additionally, we found that MALAT1 correlated negatively with VHL in HNSCC tissues, and high MALAT1/low VHL expression indicated worse prognosis of HNSCC patients, suggesting the critical role of MALAT1/VHL axis in HNSCC progression.

Then, the inhibition mechanism of MALAT1 on VHL was further explored. Previous studies have shown that MALAT1 co-localizes with SC35 nuclear speckles and performs alternative splicing of pre-mRNA [31]. Besides, MALAT1 functions as a competitive endogenous RNA to sponge miRNAs, such as miR-200c [32], miR-30a [33], miR-205 [34]. Recently, nuclear MALAT1 has been verified to influence gene expression by binding PRC2 subunits. Notably, MALAT1 recruits EZH2 (Enhancer of zeste homolog 2) and increases H3K27 trimethylation level at target loci to inhibit gene expression, thereby facilitates tumor malignancy [13]. Besides, EZH2 plays a critical role in HNSCC progression [35]. As expected, we found that the silence of EZH2, resembling si-MALAT1 effect on VHL, upregulated the expression of VHL. Although MALAT1 knockdown reduced the level of EZH2-mediated H3K27me3, no obvious enrichment of H3K27me3 was detected in the promoter of VHL in HNSCC cells. Therefore, the inhibition of EZH2 on VHL may be independent of H3K27me3 modification. Then, we found that EZH2 silencing could impair the activation of STAT3 and Akt pathways, which is in line with previous studies [36, 37]. Furthermore, the expression of VHL was increased when these two pathways were inactivated, suggesting that EZH2 downregulated VHL by activating STAT3 and Akt pathways. Thus, MALAT1 represses VHL through a non-canonical role of EZH2.

Although the role and mechanism of MALAT1 have been widely explored in HNSCC as previously described [11], lots of novel and important findings were uncovered in this study as follows: 1. In HNSCC, MALAT1 could activate NF-κB and β-catenin pathway at the posttranslational level, in which the degradation of P65/β-catenin was repressed in a VHL-dependent manner; 2. MALAT1 suppressed VHL by regulating EZH2; 3. In view of no obvious ChIP-seq signal at the gene locus of *VHL*, H3K27me3 might have little effect on the expression of VHL in HNSCC; 4. EZH2 inhibited VHL by manipulating signal transduction instead of canonical manner of histone methyltransferase; 5. Detailed studies by us and others [38–40] indicated that MALAT1-EZH2-VHL-HIF1A/P65/β-catenin positive feedback loop may exist; 6. High MALAT1/low VHL expression significantly improved the prognostic predictive capability for HNSCC patients; 7. Targeting MALAT1 dramatically inhibited the growth and even metastasis in HNSCC, revealing the translational role of MALAT1 in HNSCC treatment and the great potential of anti-MALAT1 therapy.

In summary, we identify that MALAT1 is aberrantly expressed and indicates unfavorable outcome in HNSCC. MALAT1 inhibits VHL to promote P65/β-catenin activation and malignant progression of HNSCC by strengthening EZH2/STAT3/Akt axis (Fig. 7G). The results of this study reveal a novel mechanism of MALAT1 in HNSCC progression, and suggest that MALAT1 is a potential prognostic biomarker and therapeutic target for HNSCC.

### Supplementary information

Supplementary materials

Full and uncropped western blots

Aj-checklist

## Data Availability

All data generated or analyzed during this study are included in this published article and supplementary information files.
