# Correction: LncRNA MALAT1 promotes growth and metastasis of head and neck squamous cell carcinoma by repressing VHL through a non-canonical function of EZH2

**DOI:** 10.1038/s41419-023-05941-7

**Published:** 2023-08-15

**Authors:** Yuansheng Duan, Kai Yue, Beibei Ye, Peng Chen, Jin Zhang, Qinghua He, Yue Wu, Qingchuan Lai, Hong Li, Yansheng Wu, Chao Jing, Xudong Wang

**Affiliations:** 1 Department of Maxillofacial and Otorhinolaryngological Oncology, Tianjin Medical University Cancer Institute and Hospital, National Clinical Research Center for Cancer, Key Laboratory of Cancer Prevention and Therapy, Tianjin, Tianjin’s Clinical Research Center for Cancer, Tianjin, 300060 China; 2 Department of Bone and Soft Tissue Tumors, Tianjin Medical University Cancer Institute and Hospital, Tianjin, 300060 China

**Keywords:** Head and neck cancer, Metastasis, Prognostic markers

Correction to: *Cell Death and Disease* 10.1038/s41419-023-05667-6, published online 22 February 2023

In this article, the affiliations 1, 2 and 3 have been given erroneously.

They should be read:

^1^Department of Maxillofacial and Otorhinolaryngological Oncology, Tianjin Medical University Cancer Institute and Hospital, National Clinical Research Center for Cancer, Key Laboratory of Cancer Prevention and Therapy, Tianjin, Tianjin’s Clinical Research Center for Cancer, Tianjin 300060, China

^2^Department of Bone and Soft Tissue Tumors, Tianjin Medical University Cancer Institute and Hospital, Tianjin 300060, China

^3^These authors contributed equally: Yuansheng Duan, Kai Yue, Beibei Ye, Peng Chen, Jin Zhang

email: yansheng1981@163.com; superdwell@hotmail.com; wxd.1133@163.com

The original article has been corrected.

