# Supplementary material for: LncRNA MALAT1 promotes growth and metastasis of head and neck squamous cell carcinoma by repressing VHL through a non-canonical function of EZH2
Source: Cell Death Dis. 2023 Feb 22;14(2):149. doi: 10.1038/s41419-023-05667-6 (PMC9946937; doi:10.1038/s41419-023-05667-6)
Supplement: Supplementary file 1 — Supplementary materials [file 41419_2023_5667_MOESM1_ESM.docx]

**Supplementary Table S1. List of siRNAs sequence.**

| siRNA name | Sequence (5’-3’) |
| --- | --- |
| si-MALAT1 #1 | GATCCATAATCGGTTTCAA |
| si-MALAT1 #2 | GCAAATGAAAGCTACCAAT |
| si-MALAT1 #3 | CCTCAGACAGGTATCTCTT |
| si-EZH2 #1 | GAGGTTCAGACGAGCTGAT |
| si-EZH2 #2 | GCAAATTCTCGGTGTCAAA |
| si-EZH2 #3 | GAGGGAAAGTGTATGATAA |
| si-VHL #1 | GCTCTACGAAGATCTGGAA |
| si-VHL #2 | CCGTATGGCTCAACTTCGA |
| si-VHL #3 | AGGAGCGCATTGCACATCA |

**Supplementary Table S2. Univariate analysis of clinicopathological features for overall survival.**

| **Clinicopathological Features** | **Survival status** | | **Total** | ***P* value** |
| --- | --- | --- | --- | --- |
|  | **Survival** | **Death** |  |  |
| **Age** |  |  |  | 0.284 |
| ≤45 yr | 8（32.0%） | 17（68.0%） | 25 |  |
| ＞45 yr | 60（38.8%） | 78（61.2%） | 138 |  |
| **Gender** |  |  |  | 0.276 |
| Male | 22（30.6%） | 50（69.4%） | 72 |  |
| Female | 26（39.4%） | 40（60.6%） | 66 |  |
| **Smoking** |  |  |  | 0.173 |
| No | 53（45.7%） | 63（54.3%） | 116 |  |
| Yes | 21（35.0%） | 39（65.0%） | 60 |  |
| **Drinking** |  |  |  | 0.375 |
| No | 56（44.1%） | 71（55.9%） | 127 |  |
| Yes | 18（36.7%） | 31（63.3%） | 49 |  |
| **Histological grade** |  |  |  | **0.033*** |
| High | 24（53.3%） | 21（46.7%） | 45 |  |
| Medium | 32（43.2%） | 42（56.8%） | 74 |  |
| Poor | 13（27.1%） | 35（72.9%） | 48 |  |
| **Clinical stage** |  |  |  | **0.003*** |
| I-II | 34（58.6%） | 24（41.4%） | 58 |  |
| III-IV | 39（35.1%） | 72（64.9%） | 111 |  |
| **T stage** |  |  |  | **0.013*** |
| T1-2 | 46（52.3%） | 42（47.7%） | 88 |  |
| T3-4 | 27（33.3%） | 54（66.7%） | 81 |  |
| **LN metastasis** |  |  |  | **0.002*** |
| negative | 48（54.5%） | 40（45.5%） | 88 |  |
| positive | 25（30.9%） | 56（69.1%） | 81 |  |
| **MALAT1 expression** |  |  |  | **0.002*** |
| Low | 28（50.0%） | 28（50.0%） | 56 |  |
| High | 20（24.4%） | 62（75.6%） | 82 |  |
| **NOTE:** The result was analyzed by the Pearson χ2 test. *P* values with significance were shown as asterisk. *, *P*<0.05. | | | | |

**Supplementary Table S3. Multivariate Cox regression analysis of risk factors for overall survival.**

| **Clinicopathological Features** | **B** | **S.E.** | **Wald** | **DF** | ***P* Value** | **OR** | **95% C.I.for OR** | |
| --- | --- | --- | --- | --- | --- | --- | --- | --- |
|  |  |  |  |  |  |  | **Lower** | **Upper** |
| **Histological grade** |  |  |  |  |  |  |  |  |
| High | 0.000 |  |  |  |  | 1.000 |  |  |
| Medium | 0.397 | 0.274 | 2.093 | 1.000 | 0.148 | 1.487 | 0.869 | 2.547 |
| Poor | 1.067 | 0.294 | 13.160 | 1.000 | **0.000*** | 2.907 | 1.633 | 5.174 |
| **LN metastasis** |  |  |  |  |  |  |  |  |
| Yes | 0.000 |  |  |  |  | 1.000 |  |  |
| No | 0.490 | 0.223 | 4.822 | 1.000 | **0.028*** | 1.632 | 1.054 | 2.525 |
| **MALAT1 expression** |  |  |  |  |  |  |  |  |
| Low | 0.000 |  |  |  |  | 1.000 |  |  |
| High | 0.481 | 0.221 | 4.762 | 1.000 | **0.029*** | 1.618 | 1.050 | 2.493 |
| **NOTE:** *P* values with significance were shown as asterisk. *, *P*<0.05. | | | | | | | | |

**
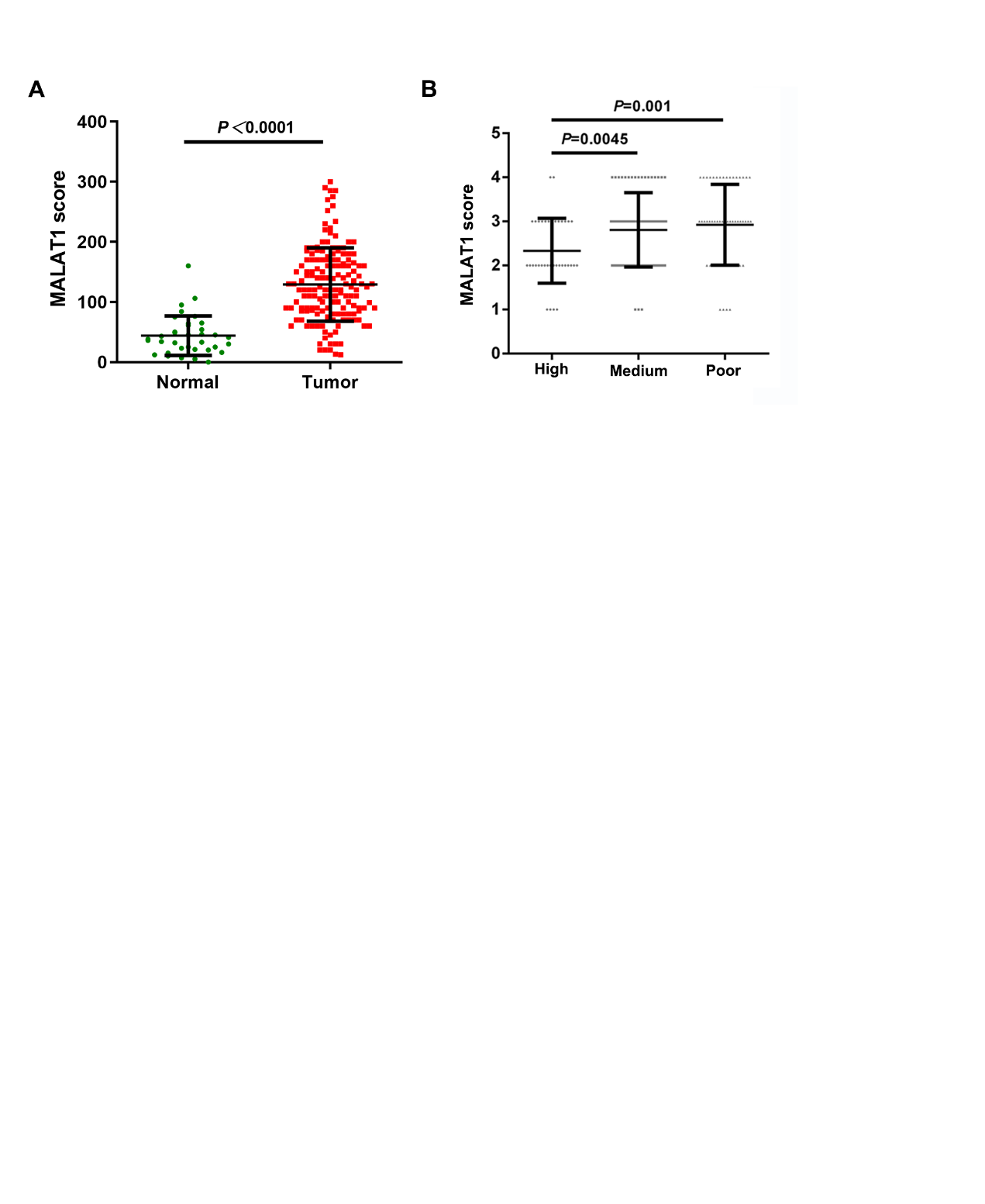
**

**Supplementary Fig. 1 MALAT1 is significantly upregulated in HNSCC specimens compared with normal tissues.** Data, mean ± SD. Normal tissues, 37 cases; Tumor samples, 157 cases.

**
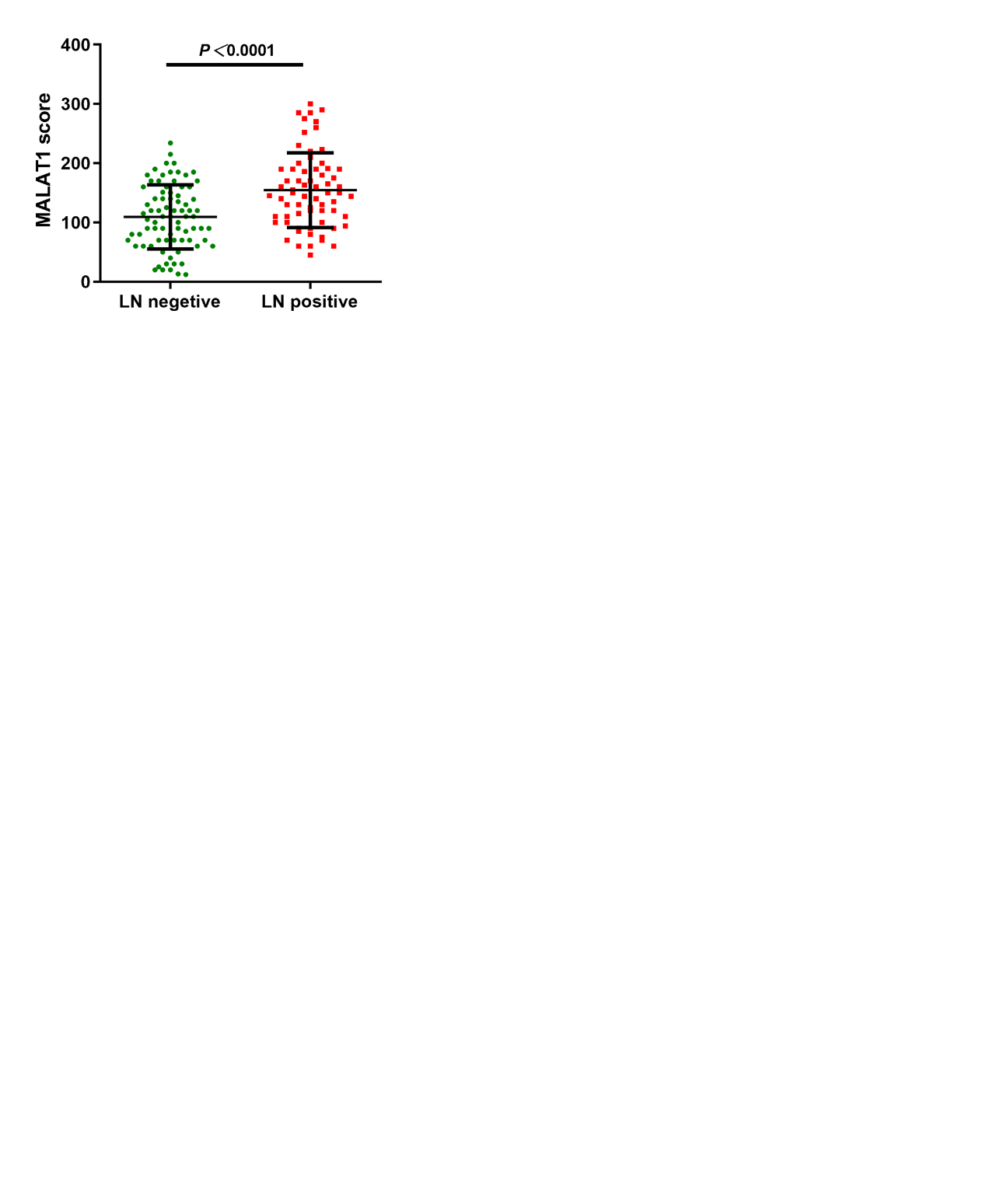
**

**Supplementary Fig. 2 MALAT1 expression positively correlates with lymph node metastasis in HNSCC.** Data, mean ± SD. LN, lymph node. Tumor with negative LN, 75 cases; Tumor with positive LN, 70 cases.

**
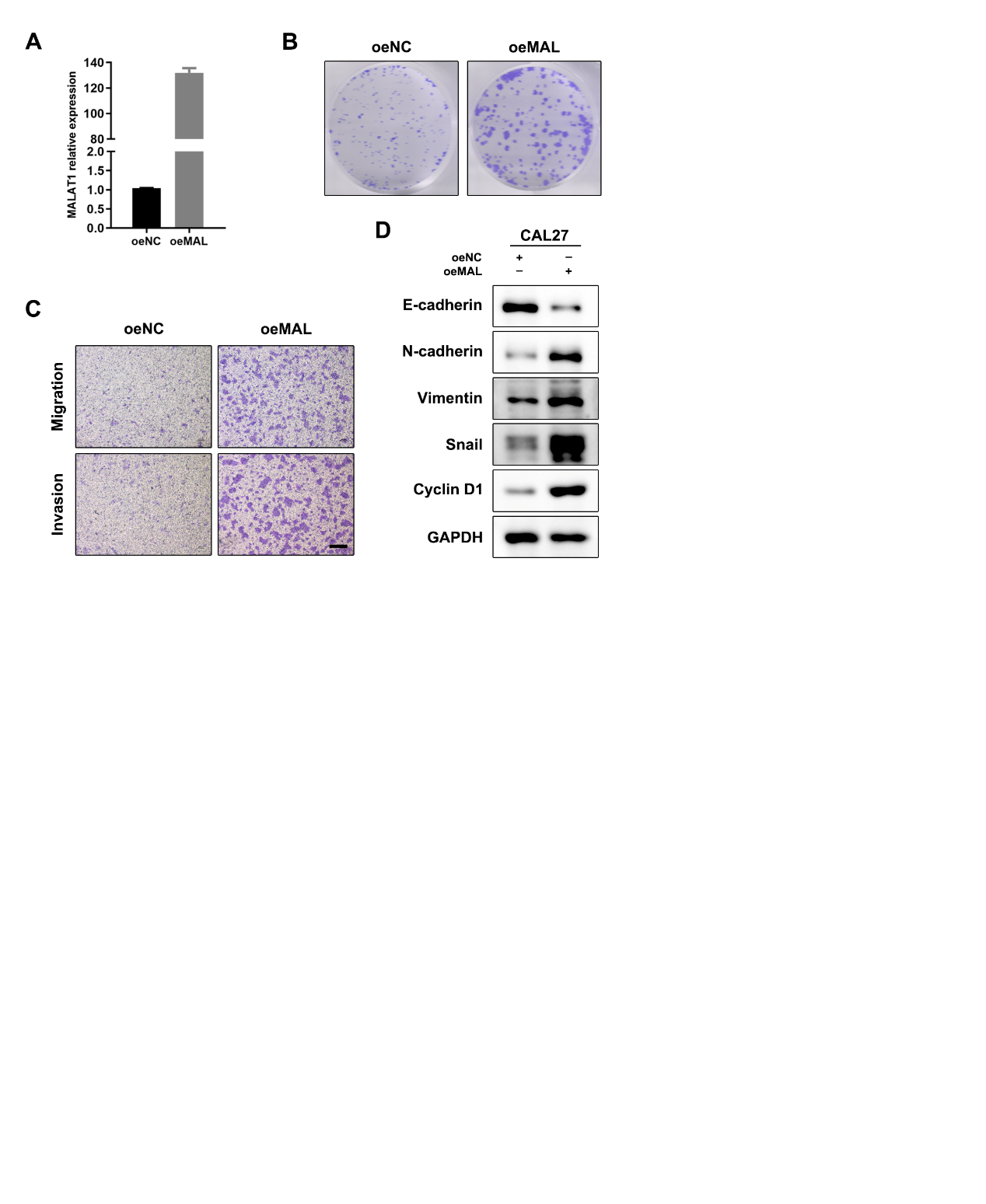
**

**Supplementary Fig. 3 MALAT1 promotes HNSCC progression *in vitro.* A** The level of MALAT1 was detected in CAL27 cells overexpressing MALAT1 by using qPCR assay. **B** The ability of colony formation was increased in CAL27 cells overexpressing MALAT1. **C** MALAT1 overexpression promoted migration and invasion in CAL27 cells. Scale bar, 100 μm. **D** Western blot assay was performed to determine the protein level of E-cadherin, N-cadherin, Vimentin, Snail and Cyclin D1 in CAL27 cells overexpressing MALAT1. Data in this figure, mean ± SD. oeNC, negative control for overexpression. oeMAL, MALAT1 overexpression.


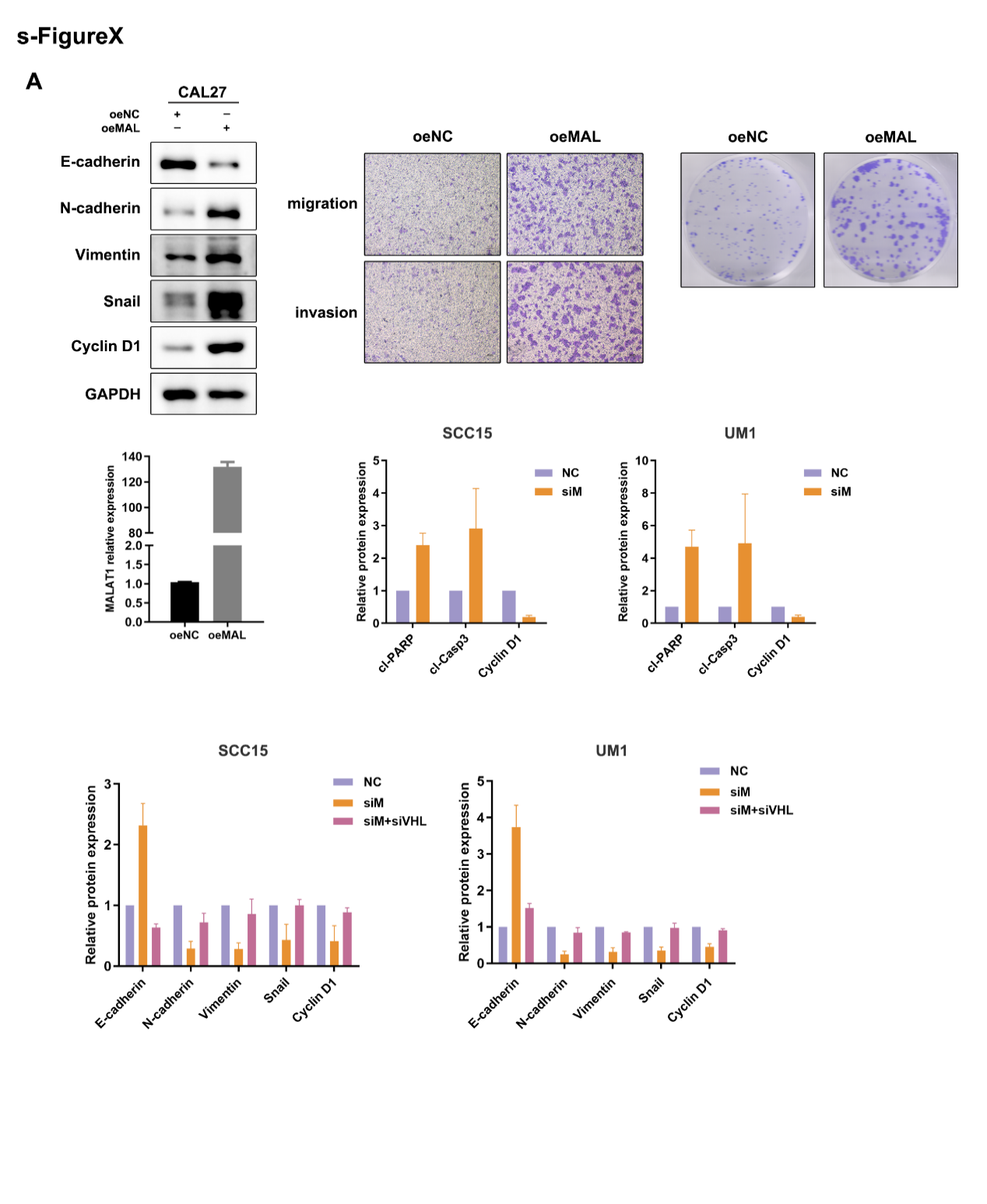


**Supplementary Fig. 4 Quantification by densitometry was performed according to the results shown in Figure 2G.** The WB assay was repeated three times. Data, mean ± SD. NC, negative control. siM, si-MALAT1. cl-PARP, cleaved PARP. cl-Casp3, cleaved Caspase-3.


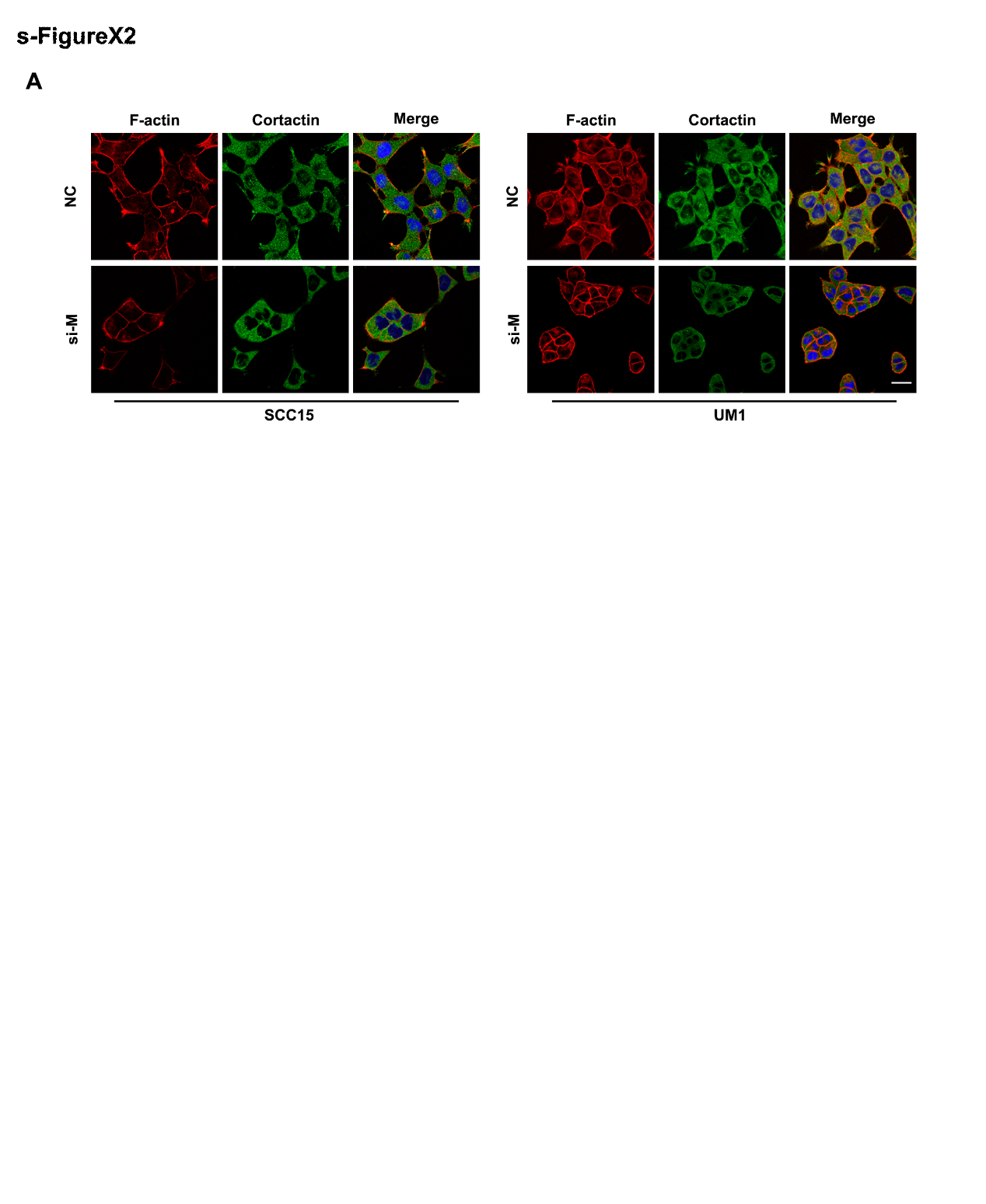


**Supplementary Fig. 5 MALAT1 promotes the formation of invadopodia in UM1 cells.** Representative images of immunofluorescence of F-actin and Cortactin in indicated groups were shown. Scale bar, 20 μm. NC, negative control. si-M, si-MALAT1.


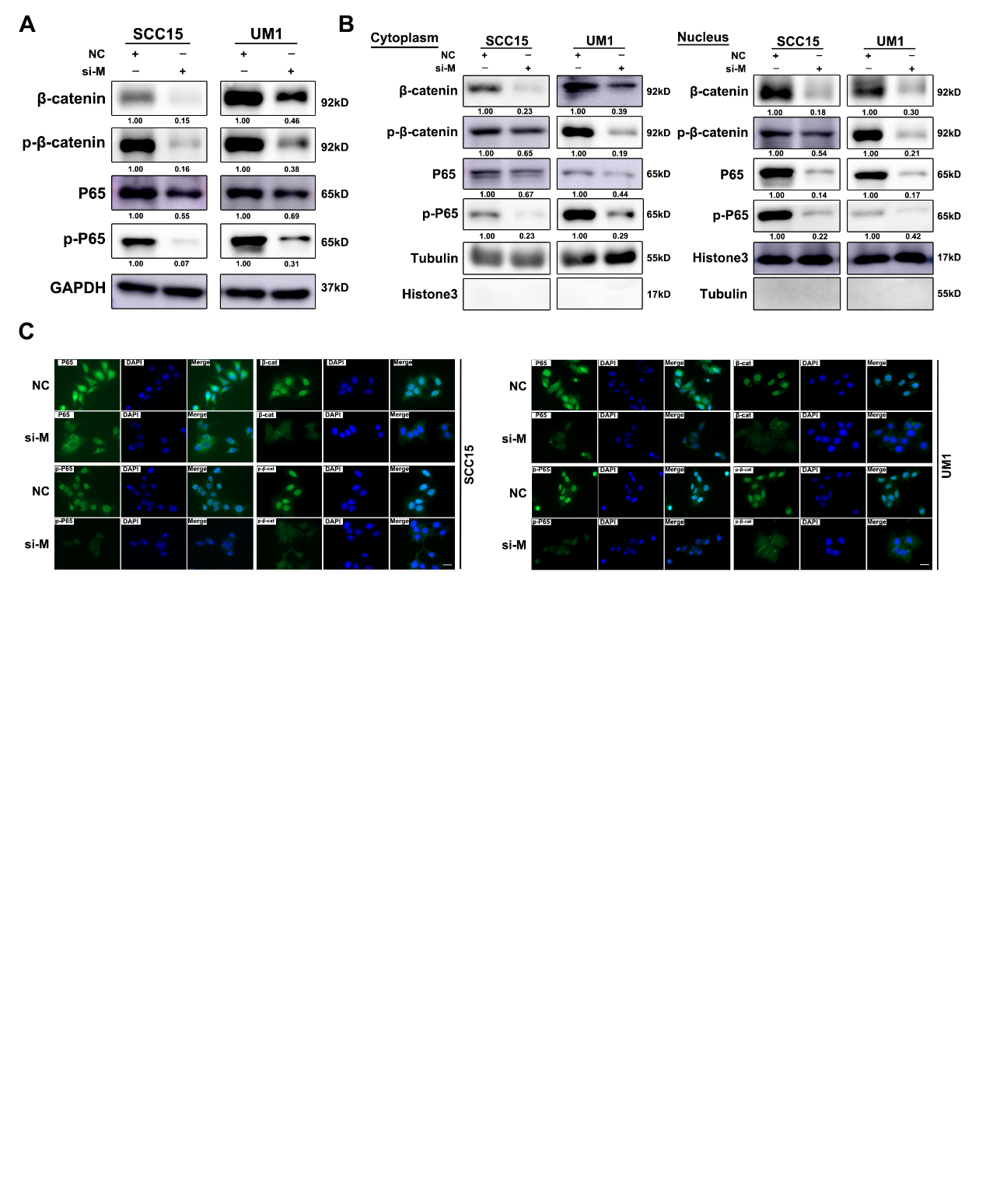


**Supplementary Fig. 6 Silence of MALAT1 inhibits the activation of β-catenin and NF-κB pathways. A** The abundance of β-catenin, p-β-catenin, P65 and p-P65 was probed in SCC15 and UM1 cells transfected with si-MALAT1. **B** The immunoblotting analysis of cell fractionation indicated that MALAT1 knockdown reduced the levels of β-catenin, p-β-catenin, P65 and p-P65 in the cytoplasm and nucleus. Tubulin (for cytoplasm) and Histone3 (for nucleus) were served as internal controls, respectively. **C** The immunofluorescence staining suggested that si-MALAT1 impaired the nuclear accumulation of β-catenin, p-β-catenin, P65 and p-P65. Scale bar, 20 μm. NC, negative control. si-M, si-MALAT1.


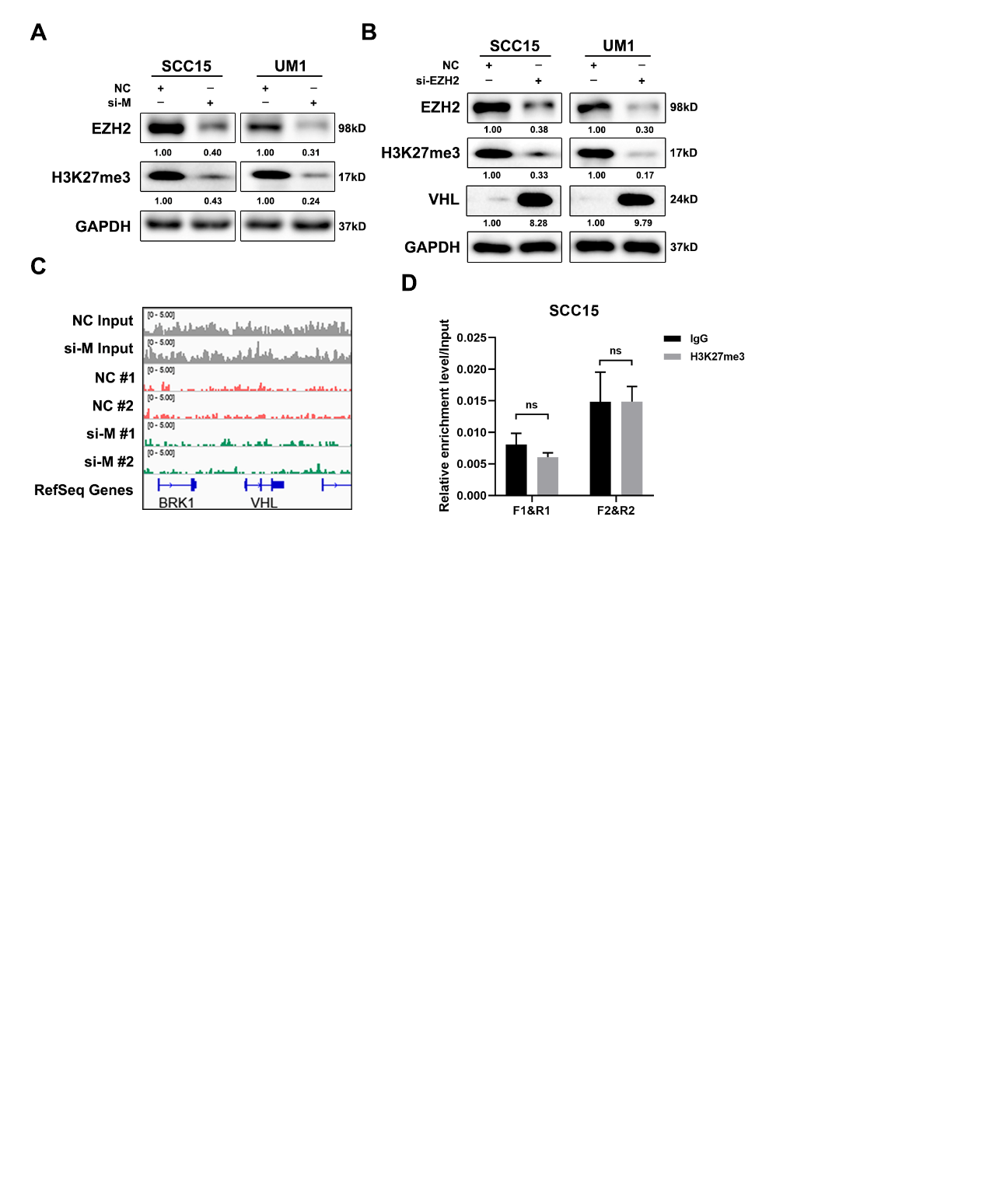


**Supplementary Fig. 7 EZH2-mediated H3K27me3 is not required for MALAT1-induced inhibition of VHL. A** The abundance of EZH2 and H3K27me3 was probed in HNSCC cells transfected with MALAT1 siRNAs. **B** The results of western blot showed that EZH2 depletion reduced the tri-methylation of H3K27 and upregulated VHL. **C** Snapshot of H3K27me3 ChIP-Seq signal at the gene locus of *VHL* in control and si-MALAT1-transfected SCC15 cells. **D** The ChIP-qPCR results indicated that no significant enrichment of H3K27me3 was found in the promoter of VHL. Two pairs of qPCR primers were used for detection. F1, forward primer 1. R1, reverse primer 1. F2, forward primer 2. R2, reverse primer 2. Data, mean ± SD. ns, no significance. NC, negative control. si-M, si-MALAT1.


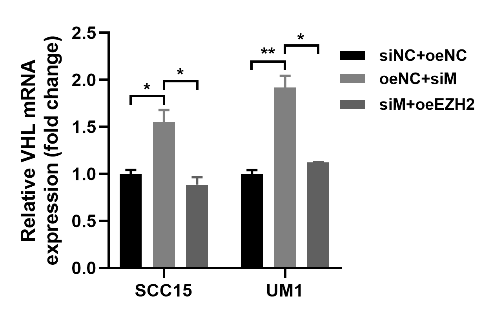


**Supplementary Fig. 8 EZH2 overexpression mitigates the inhibition of siMALAT1 on VHL mRNA.** Data, mean ± SD, **P*<0.05, ***P*<0.01. siNC, negative control for siRNA. oeNC, negative control for overexpression. siM, si-MALAT1. oeEZH2, EZH2 overexpression.


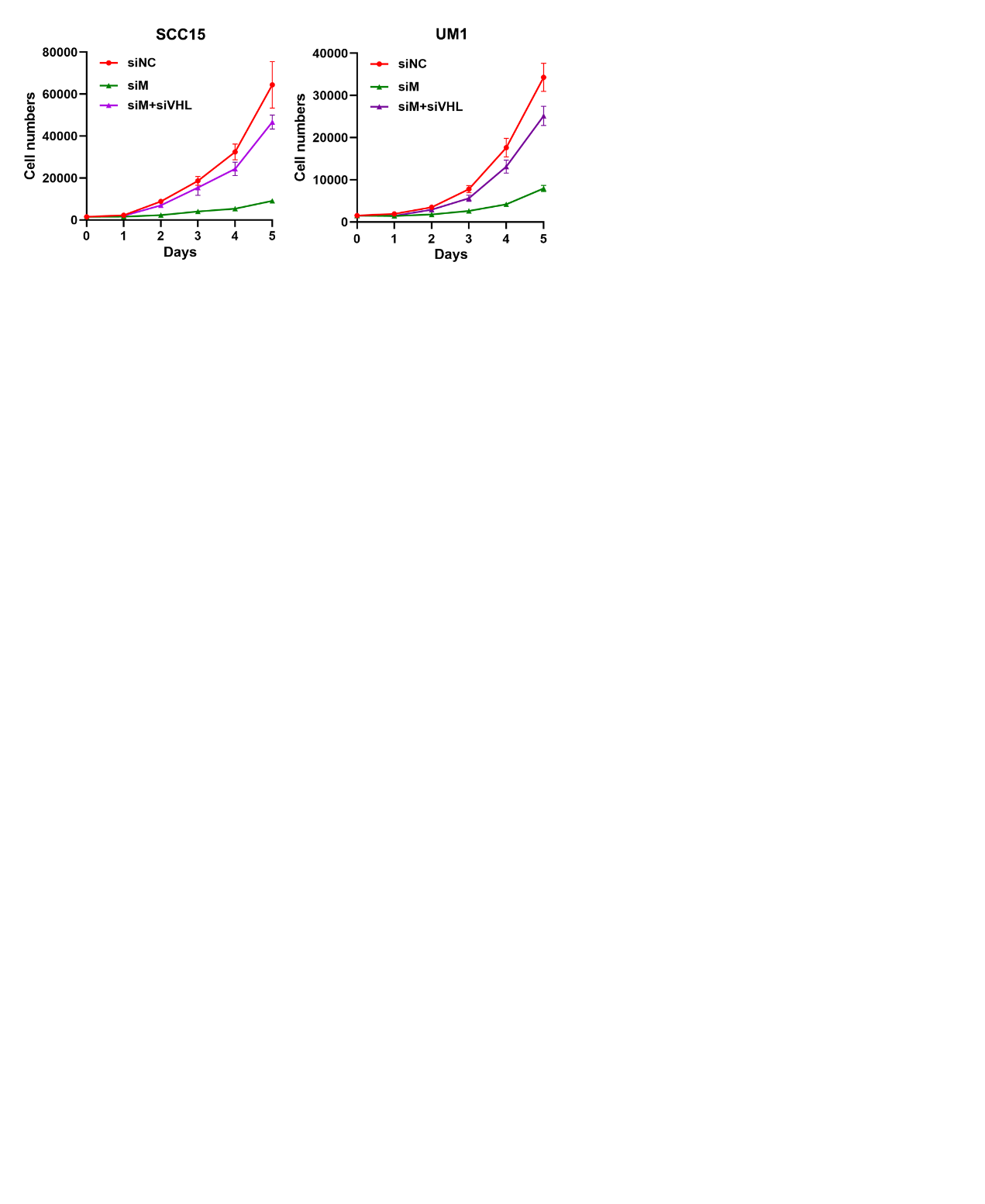


**Supplementary Fig. 9 VHL knockdown rescues the inhibition of si-MALAT1 on proliferation in HNSCC cells.** Data in this figure, mean ± SD. siNC, negative control for siRNA. siM, si-MALAT1.

**
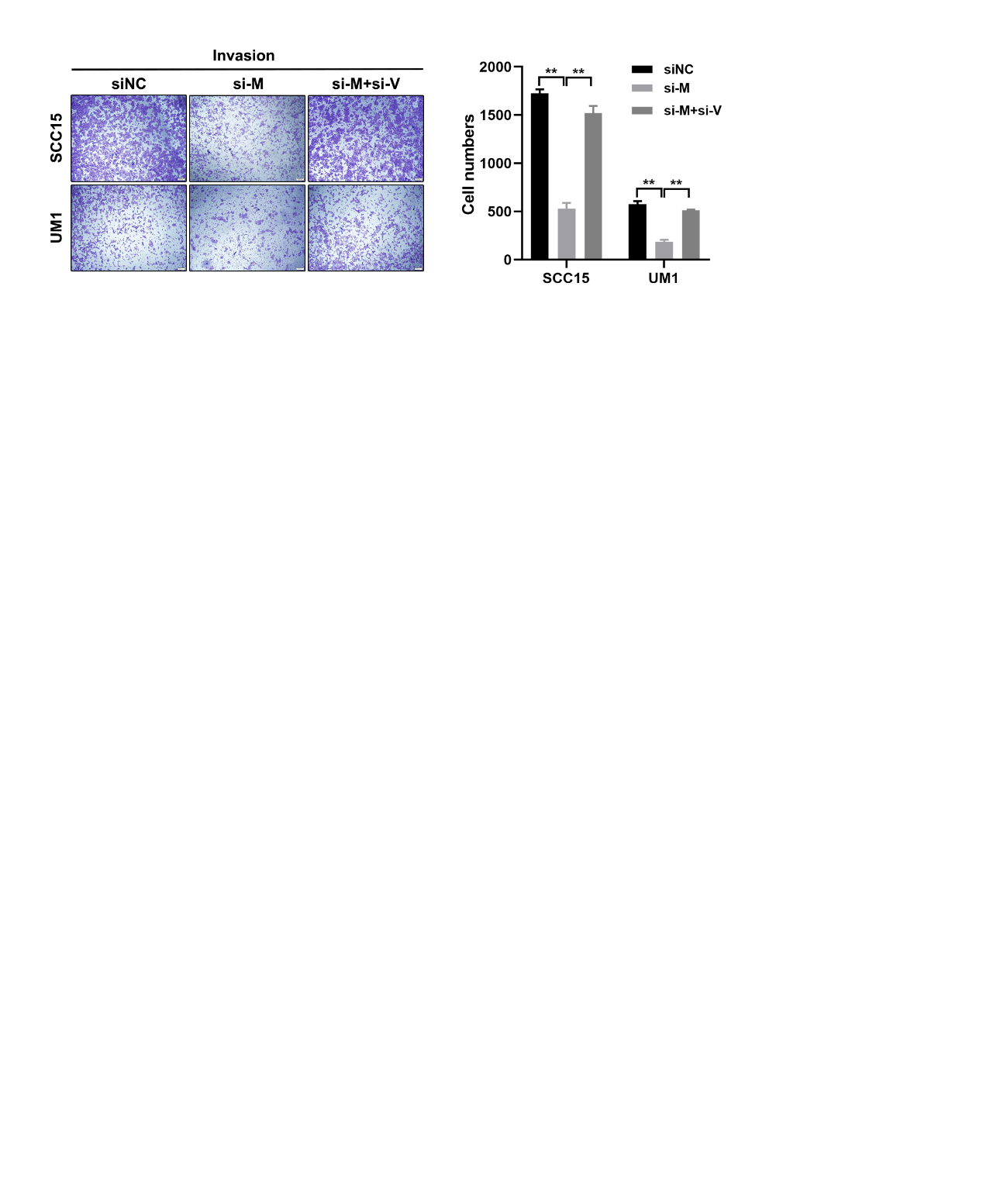
**

**Supplementary Fig. 10 Depletion of VHL restores the invasive capacity of HNSCC cells impaired by si-MALAT1.** The transwell assay was conducted in indicated groups to assess the ability of invasion. Scale bar, 100 μm. Data, mean ± SD, ***P*<0.01. siNC, negative control for siRNA. si-M, si-MALAT1. si-V, si-VHL.


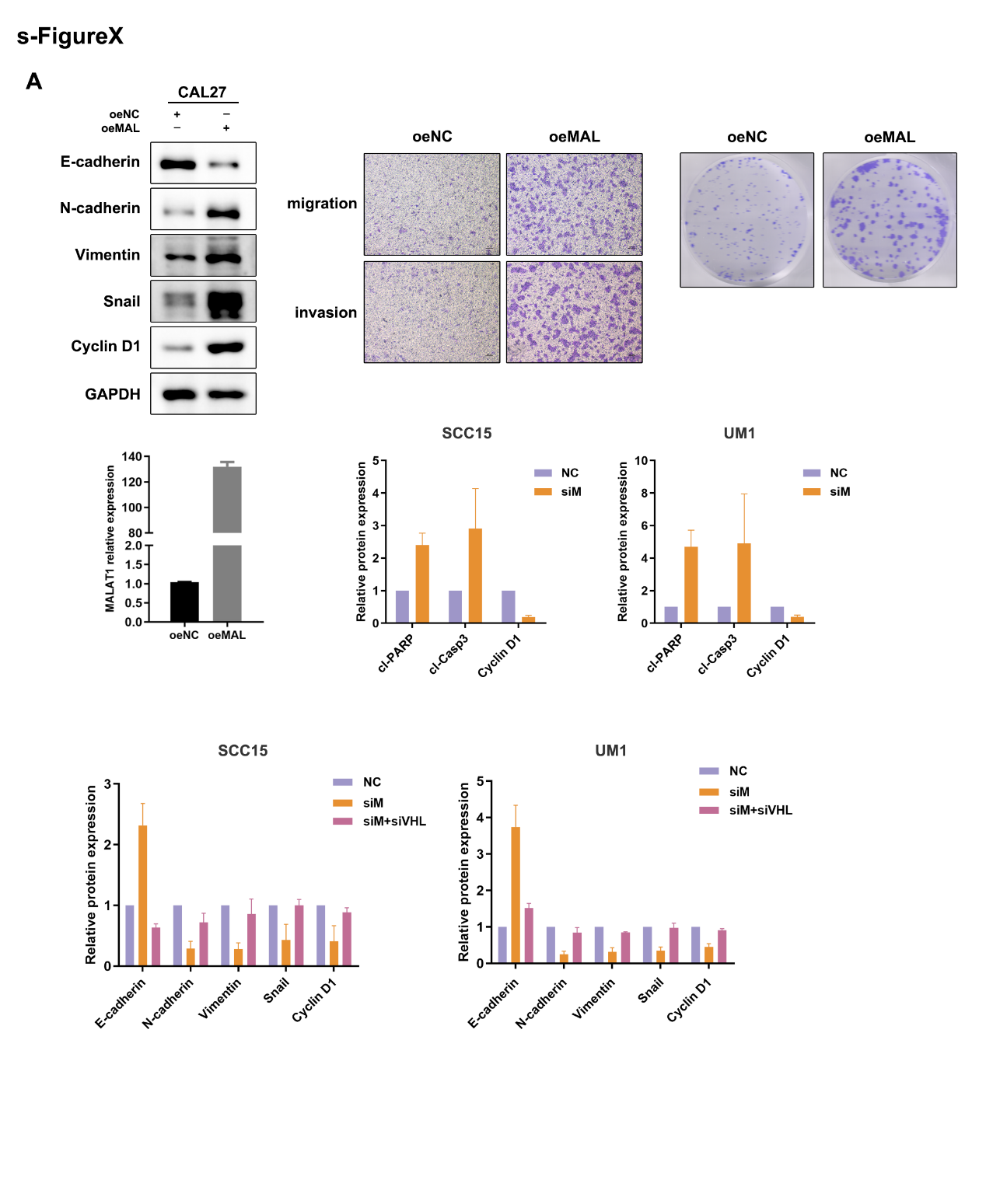


**Supplementary Fig. 11 Quantification by densitometry was performed according to the results shown in Figure 5B.** The WB assay was repeated three times. Data, mean ± SD. NC, negative control. siM, si-MALAT1.


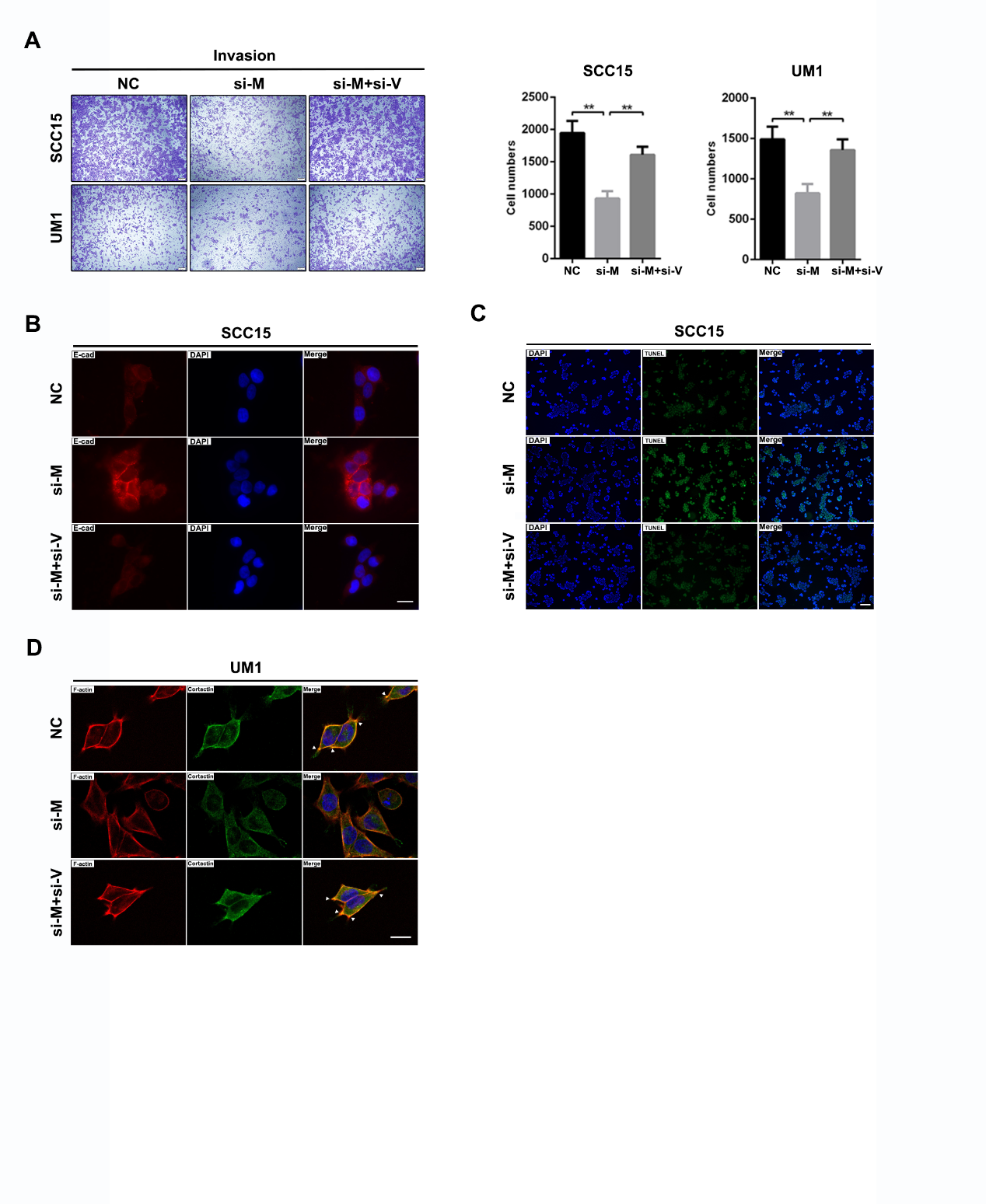


**Supplementary Fig. 12 VHL silencing inhibits the si-MALAT1-induced increase of E-cadherin.** The immunofluorescence staining of E-cadherin in SCC15 cells transfected with si-MALAT1 or si-MALAT1/si-VHL was shown, respectively. Scale bar, 20 μm. NC, negative control. si-M, si-MALAT1. si-V, si-VHL.


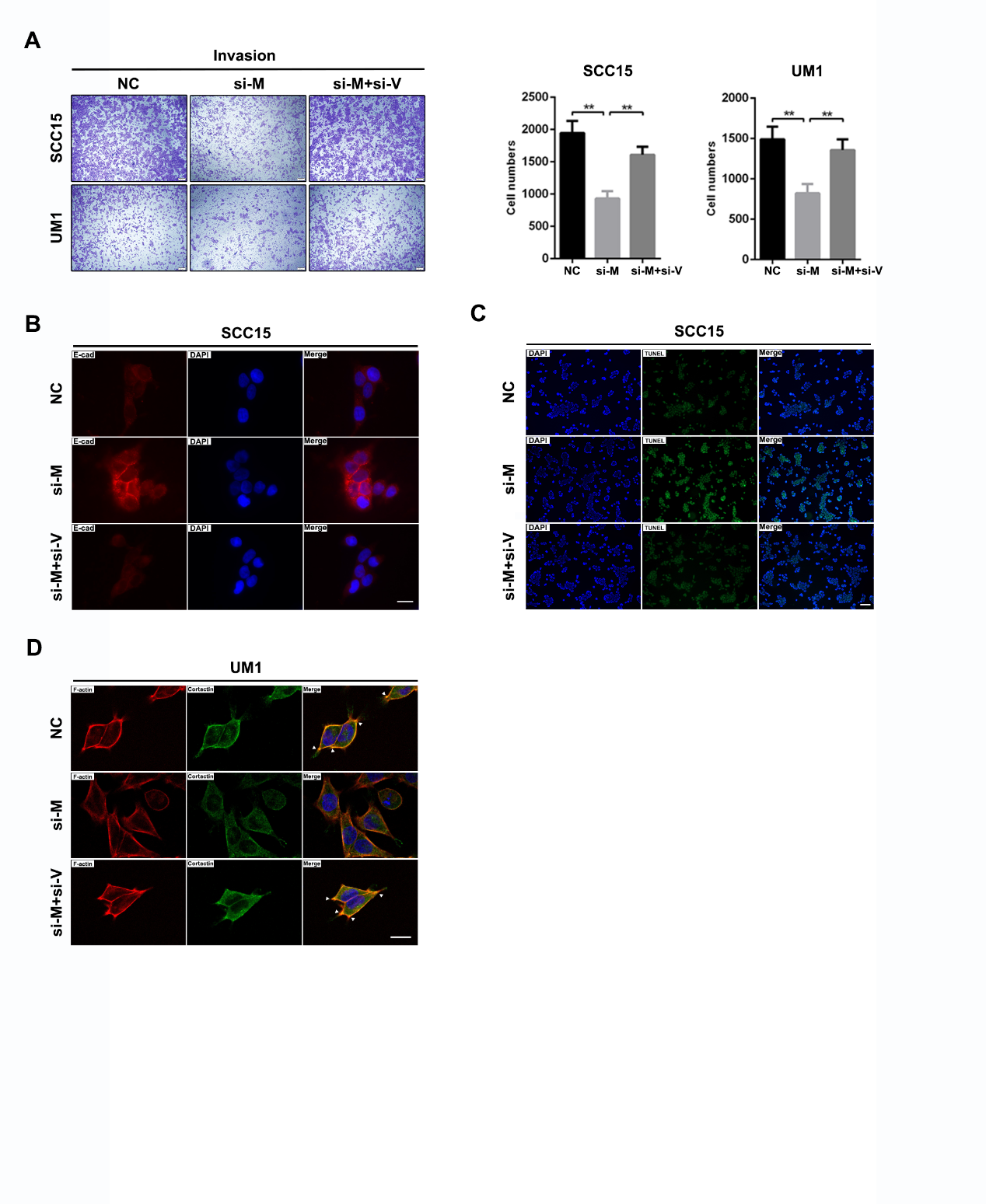


**Supplementary Fig. 13 VHL knockdown suppresses apoptosis in MALAT1-depleted SCC15 cells.** The SCC15 cells were transfected with si-MALAT1 or si-MALAT1/si-VHL, then the TUNEL assay was performed. Scale bar, 100 μm. NC, negative control. si-M, si-MALAT1. si-V, si-VHL.

**
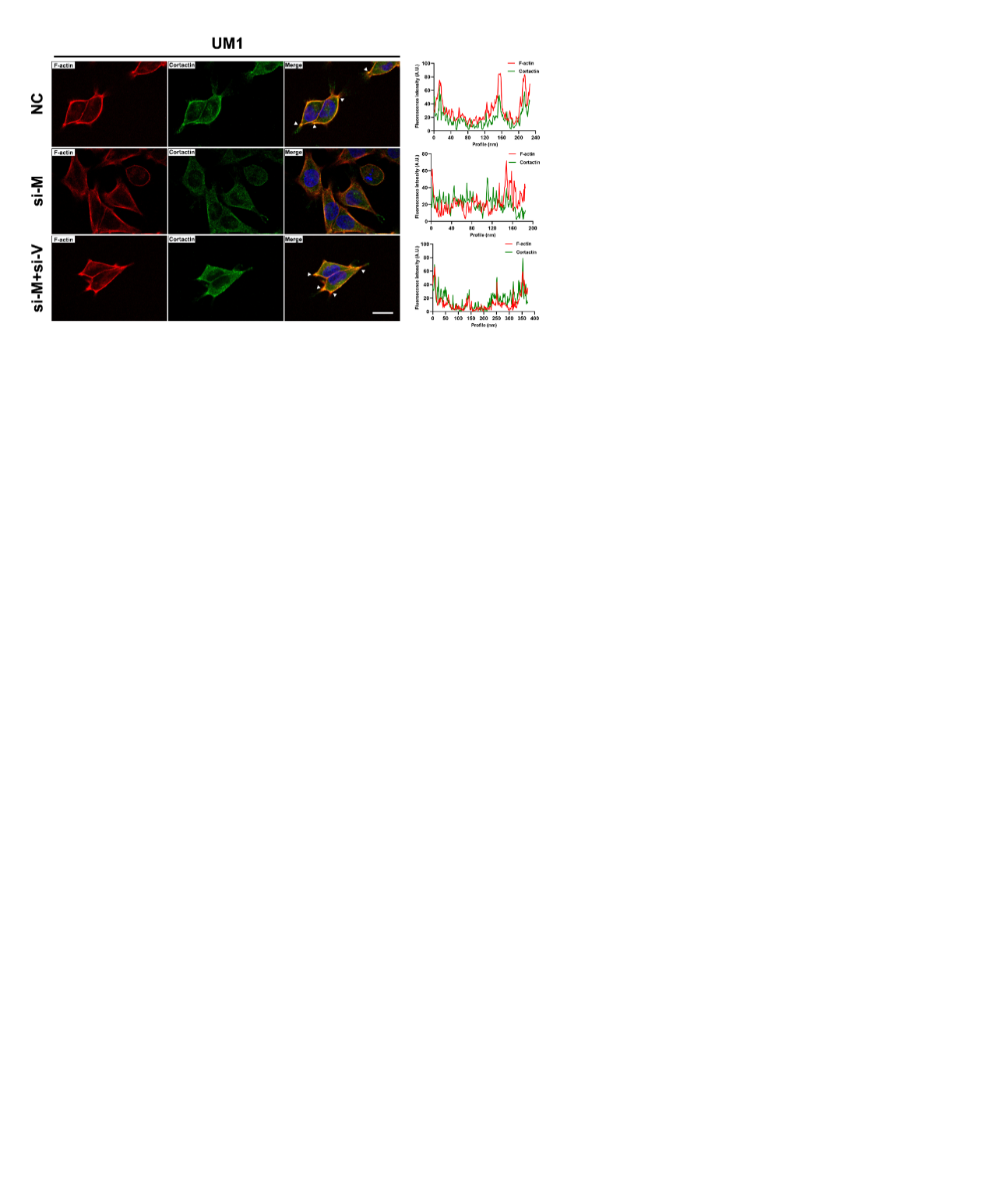
**

**Supplementary Fig. 14 VHL depletion restores the colocalization of F-actin and cortactin in si-MALAT1-delivered UM1 cells.** Representative images of immunofluorescence of F-actin and cortactin were shown. Scale bar, 20 μm. NC, negative control. si-M, si-MALAT1. si-V, si-VHL.


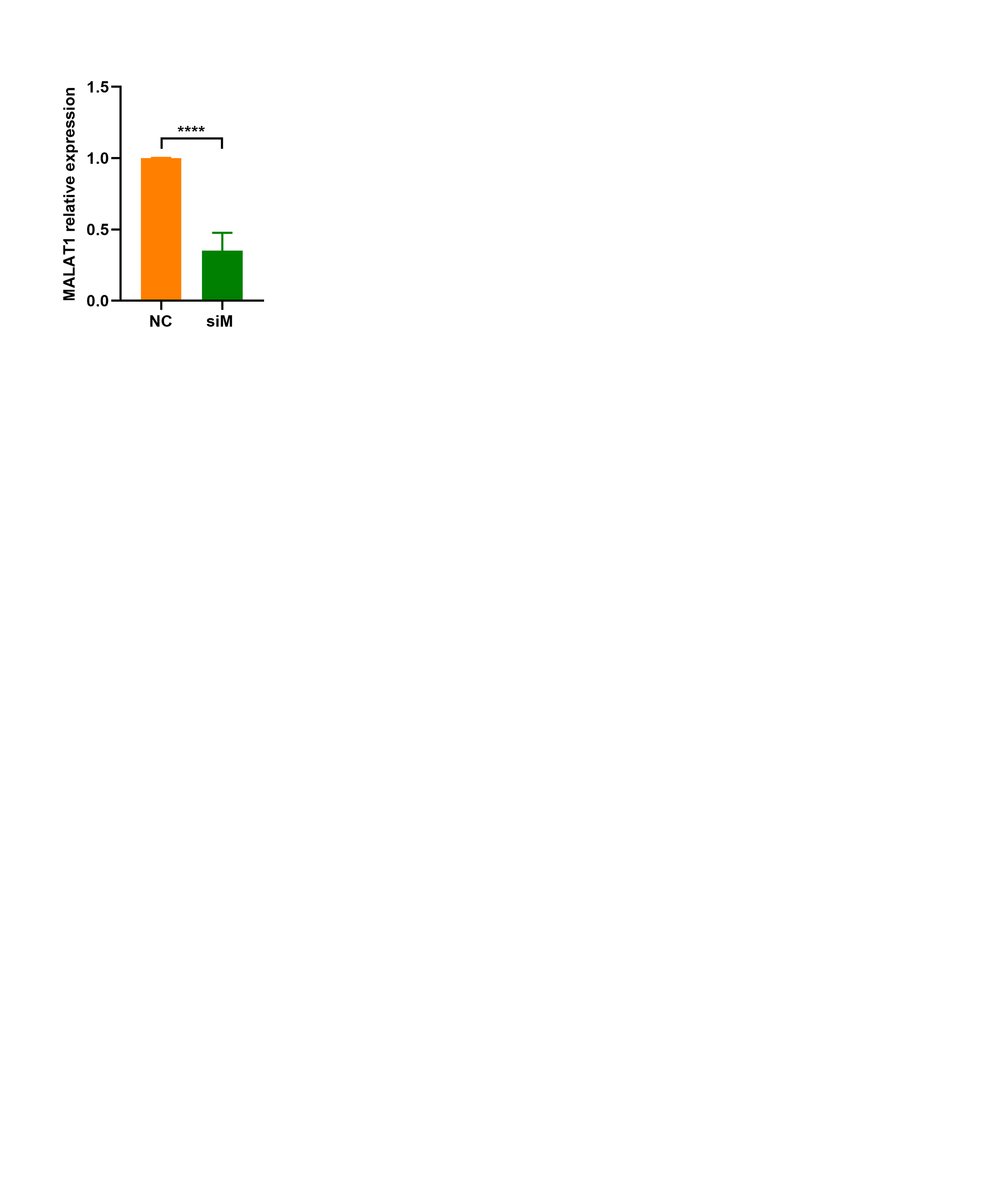


**Supplementary Fig. 15 The *in vivo* siMALAT1 represses the expression of MALAT1 in HNSCC xenografts.** The level of MALAT1 in xenografts was detected by using qPCR assay. Data, mean ± SD, *****P*<0.0001. NC, negative control. si-M, si-MALAT1.
