# Supplementary material for: LncRNA MALAT1 promotes growth and metastasis of head and neck squamous cell carcinoma by repressing VHL through a non-canonical function of EZH2
Source: Cell Death Dis. 2023 Feb 22;14(2):149. doi: 10.1038/s41419-023-05667-6 (PMC9946937; doi:10.1038/s41419-023-05667-6)

Figure 2G

Cleaved-PARP

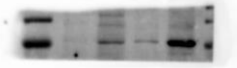

Cyclin D1

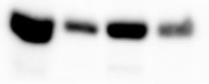

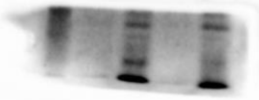

Cleaved-Caspase 3

GAPDH

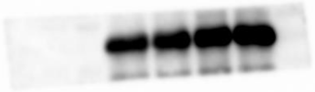

Figure 3D

E-cadherin

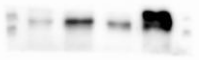

N-cadherin

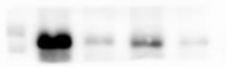

Vimentin

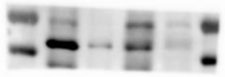

Figure 3D

Snail

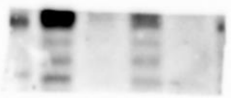

GAPDH

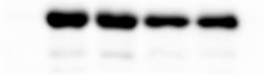

Figure 4A

4A SCC15 VHL

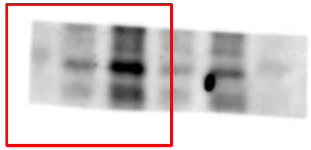

4A UM1 VHL

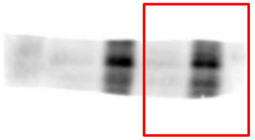

Figure 4A
S7A S7B

4A S7A GAPDH

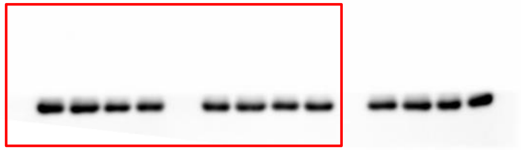

S7A SCC15 H3K27me3

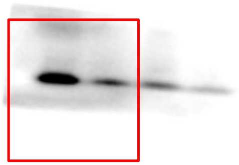

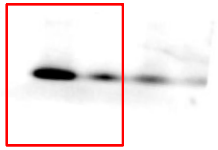

S7B SCC15 H3K27me3

S7A EZH2

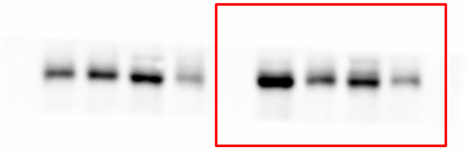

Figure S7A-B

S7A UM1 H3K27me3

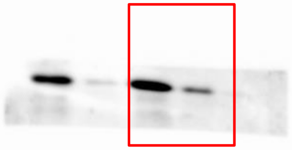

S7B UM1 H3K27me3

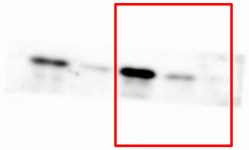

Figure S7B 4C

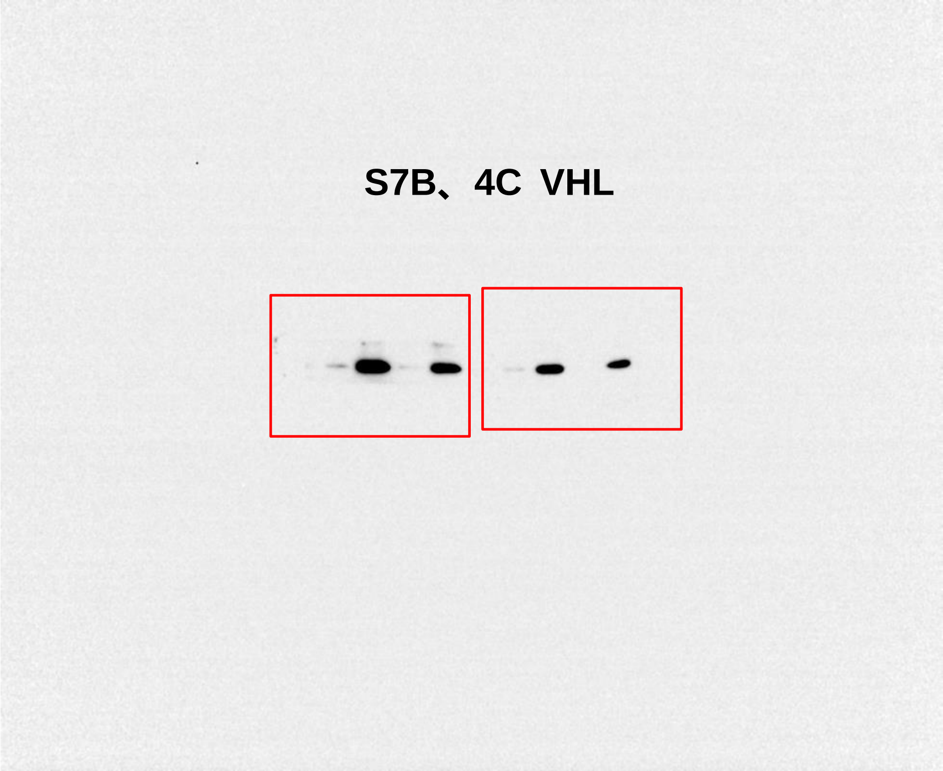

Figure S7B、4B、4C

S7B、4B、4C GAPDH

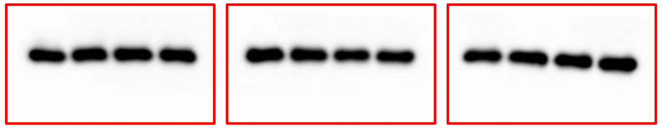

S7B、4B EZH2

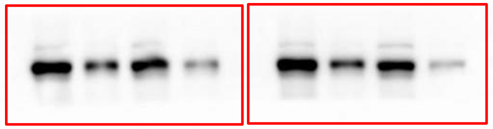

4B p-STAT3

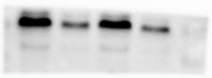

4B SCC15 STAT3

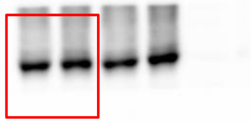

4B UM1 STAT3

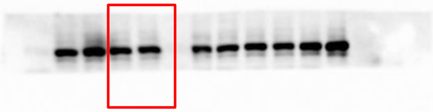

Figure 4B、4D

4B、4D p-Akt

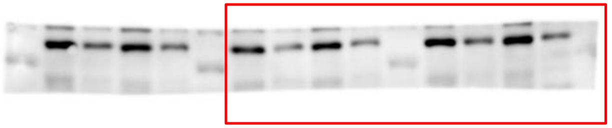

4B Akt

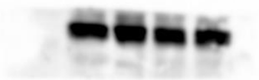

Figure 4C

4C STAT3

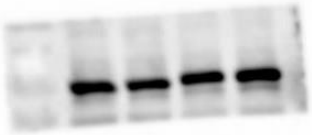

4C p-STAT3

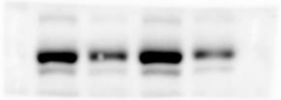

Figure 4D

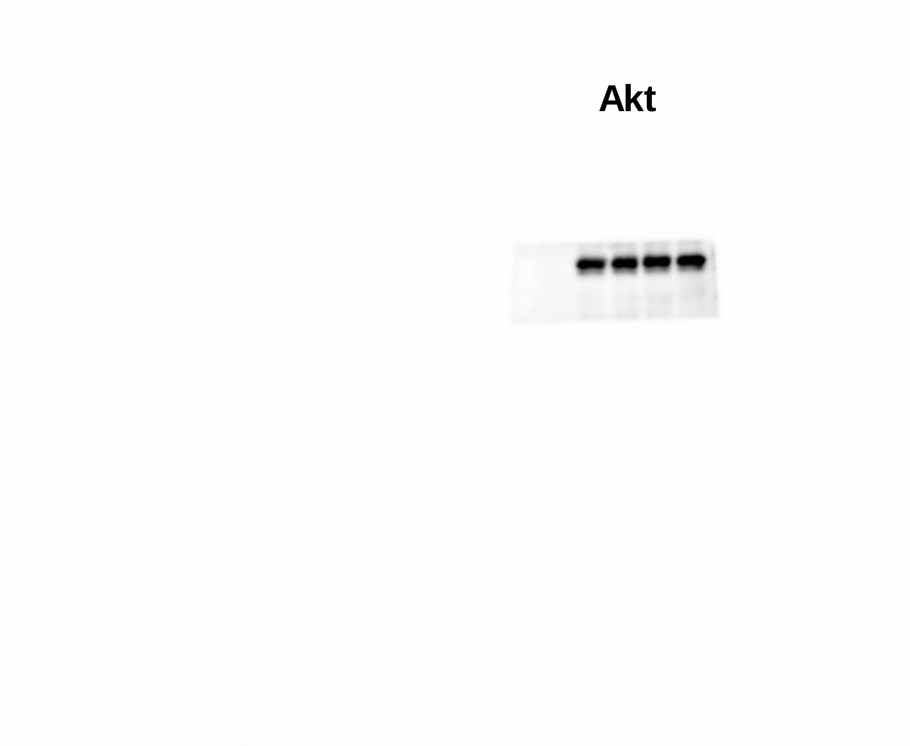

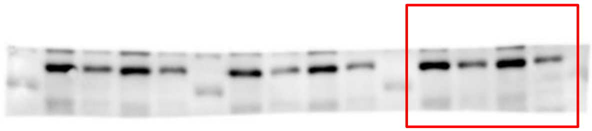

Figure 4D

4D VHL

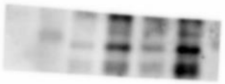

4D GAPDH

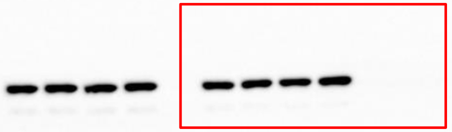

Figure 4E

UM1 EZH2

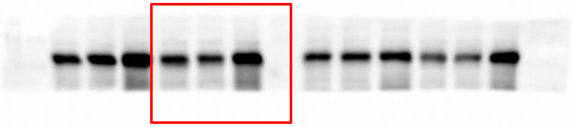

SCC15 EZH2

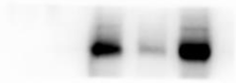

Figure 4E

SCC15 STAT3

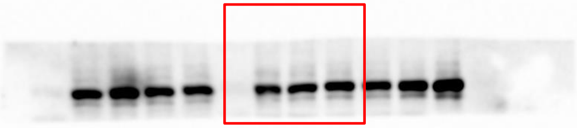

UM1 STAT3

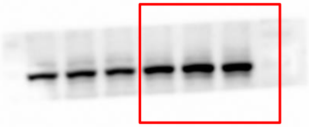

p-STAT3

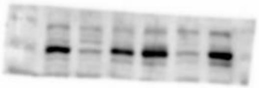

Figure 4E

UM1 AKT

SCC15 AKT

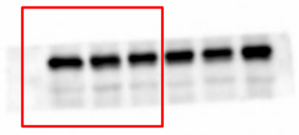

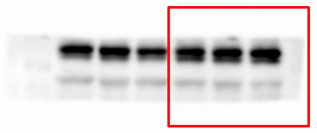

Figure 4E

SCC15 p-AKT

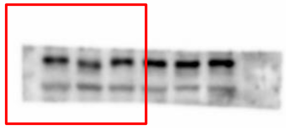

UM1 p-AKT

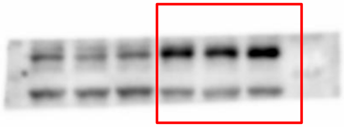

Figure 4E

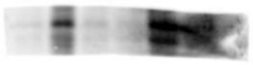

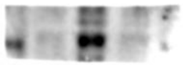

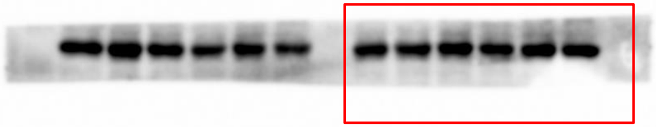

Figure 4F

VHL

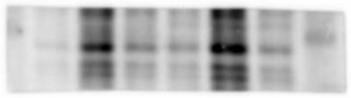

β-catenin

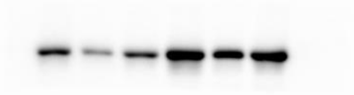

Figure 4F

p-β-catenin

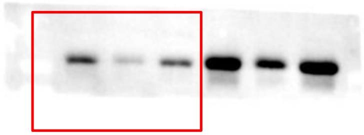

4F GAPDH

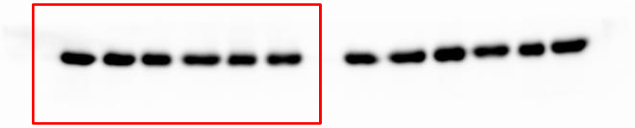

P65

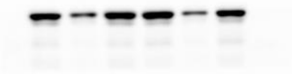

p-P65

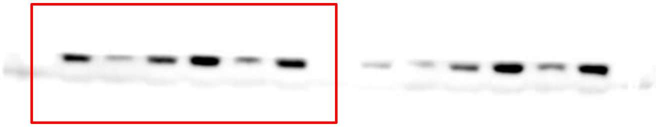

Figure 5B

5B GAPDH

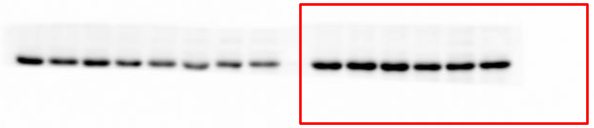

5B E-cadherin

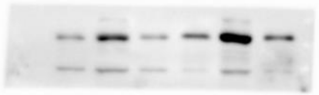

5B SCC15 N-cadherin

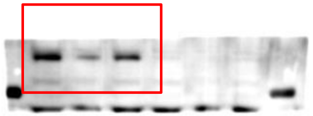

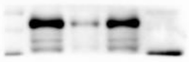

5B UM1 N-cadherin

Figure 5B

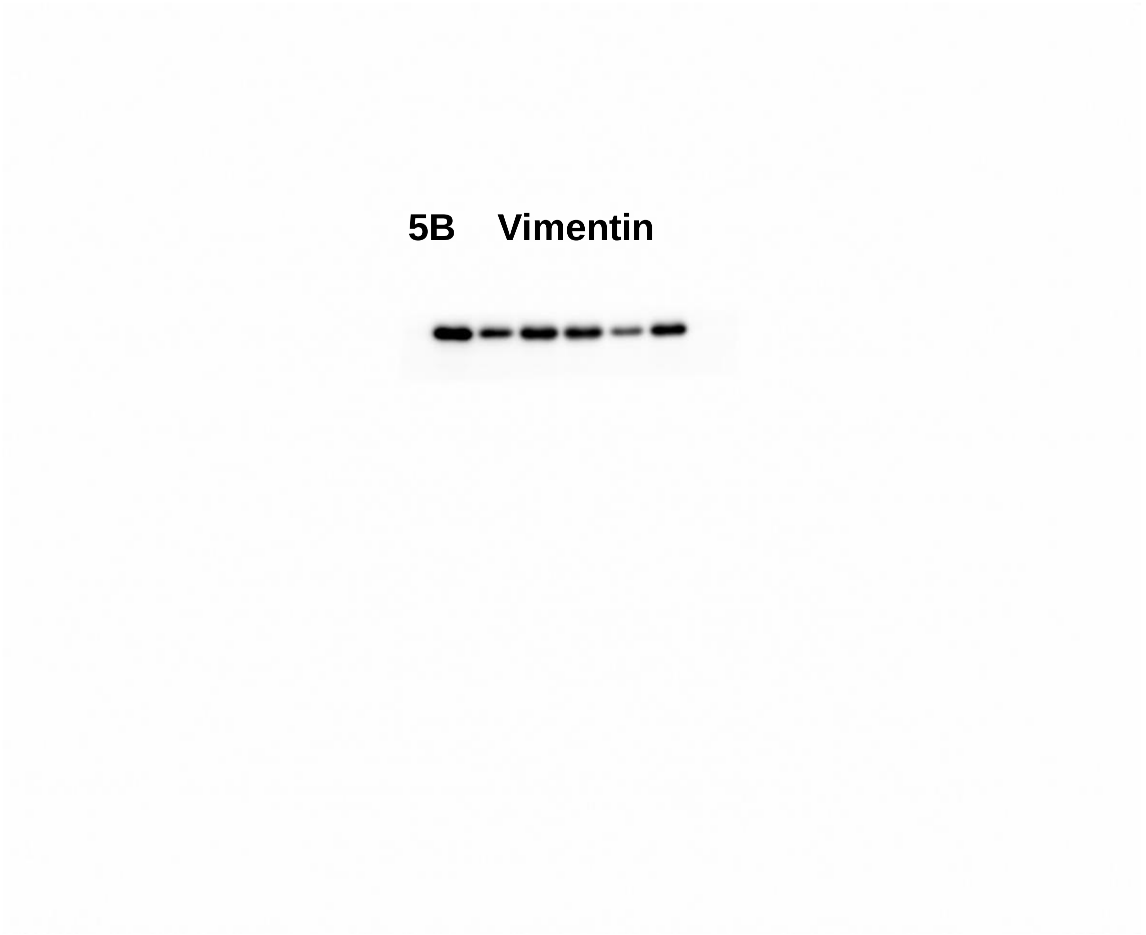

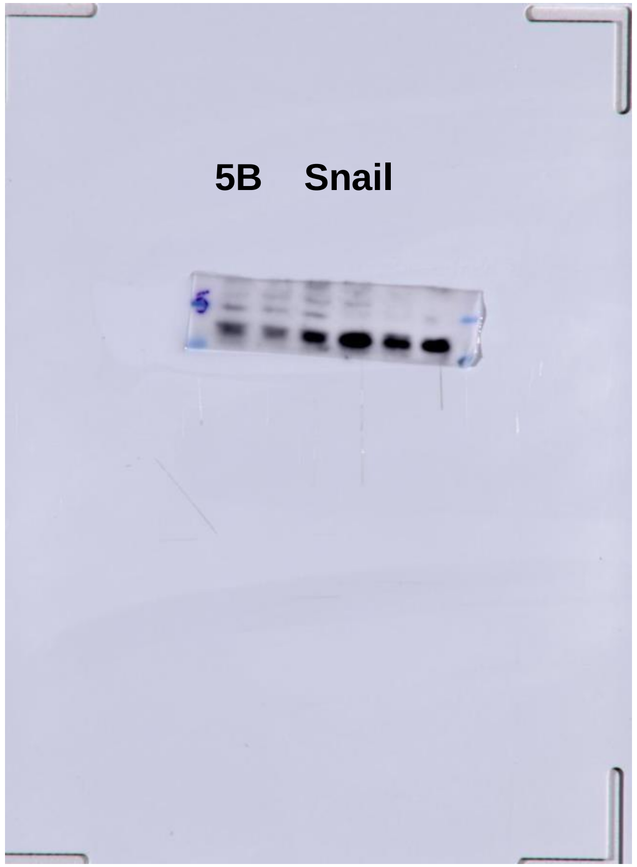

Figure 5B

5B Cyclin D1

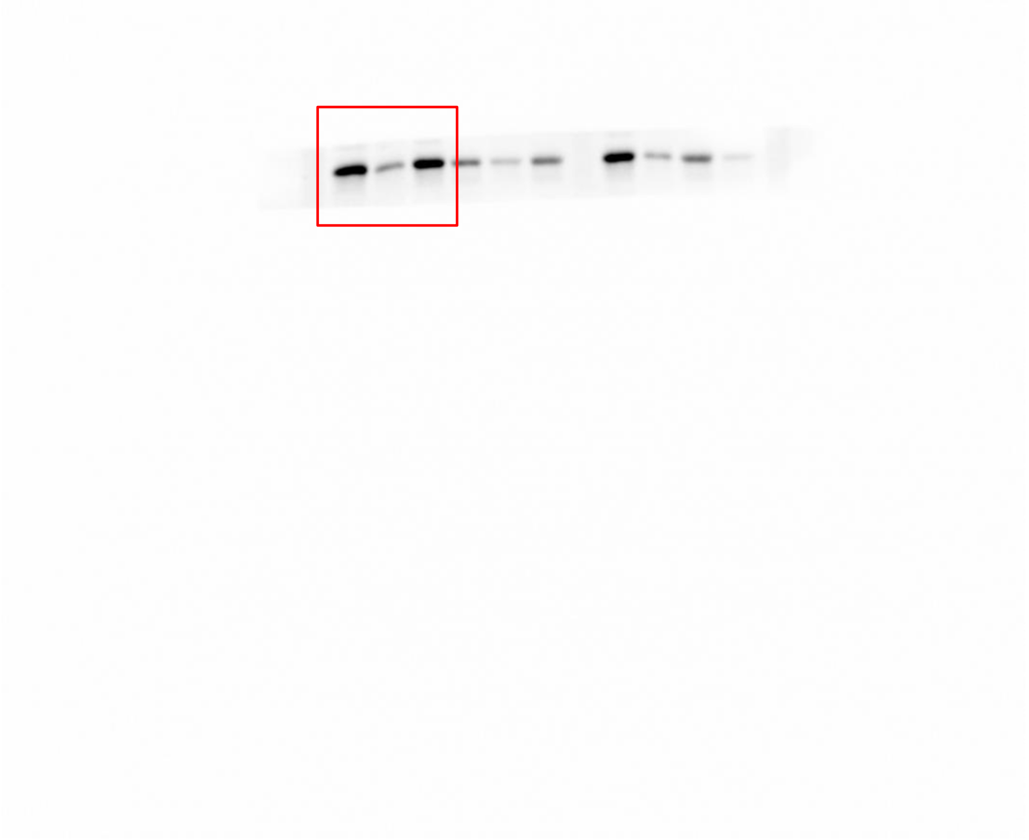

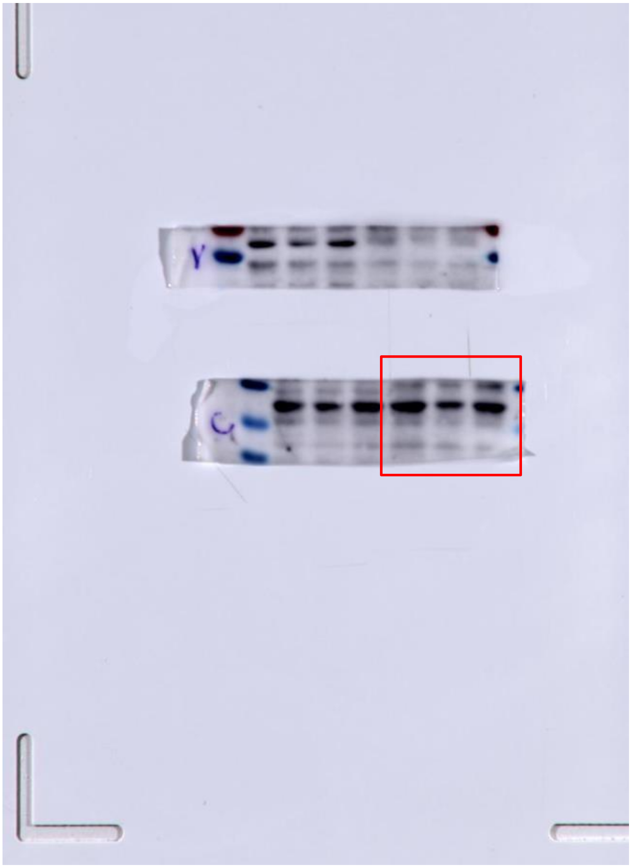

Figure S3D

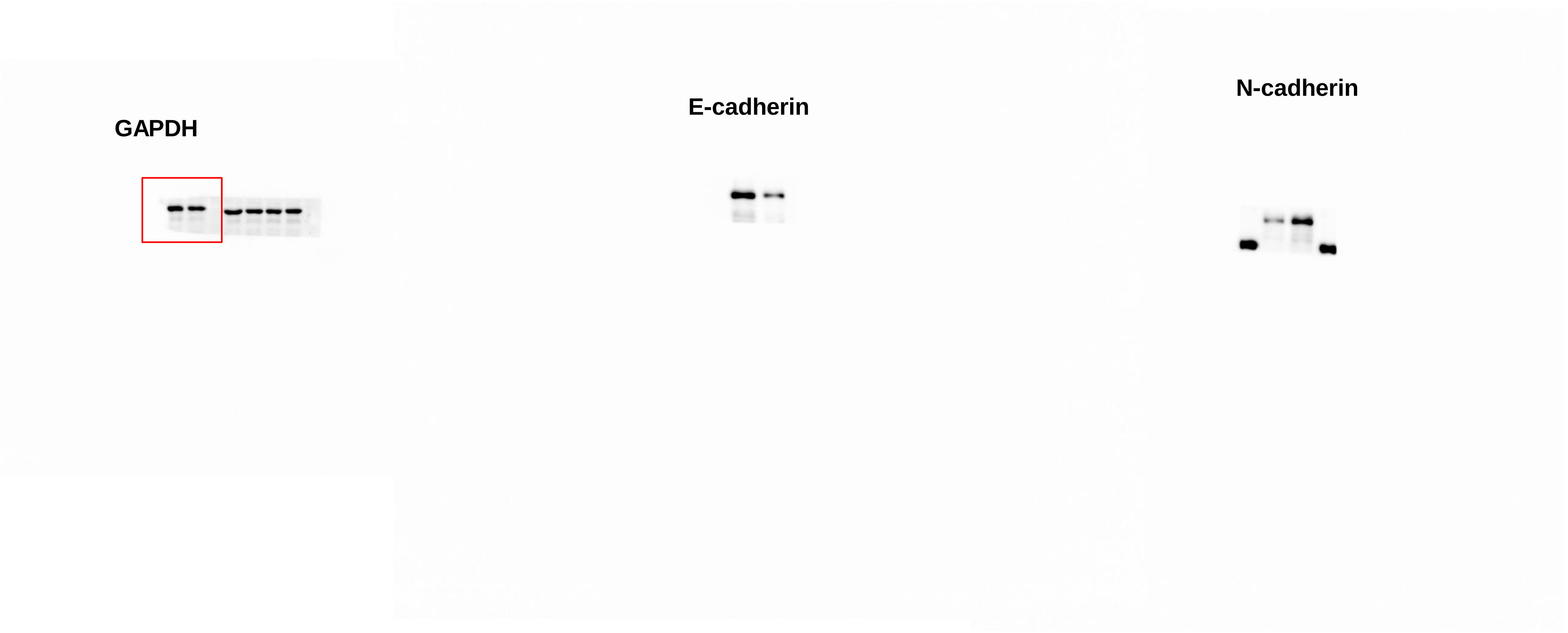

Figure S3D

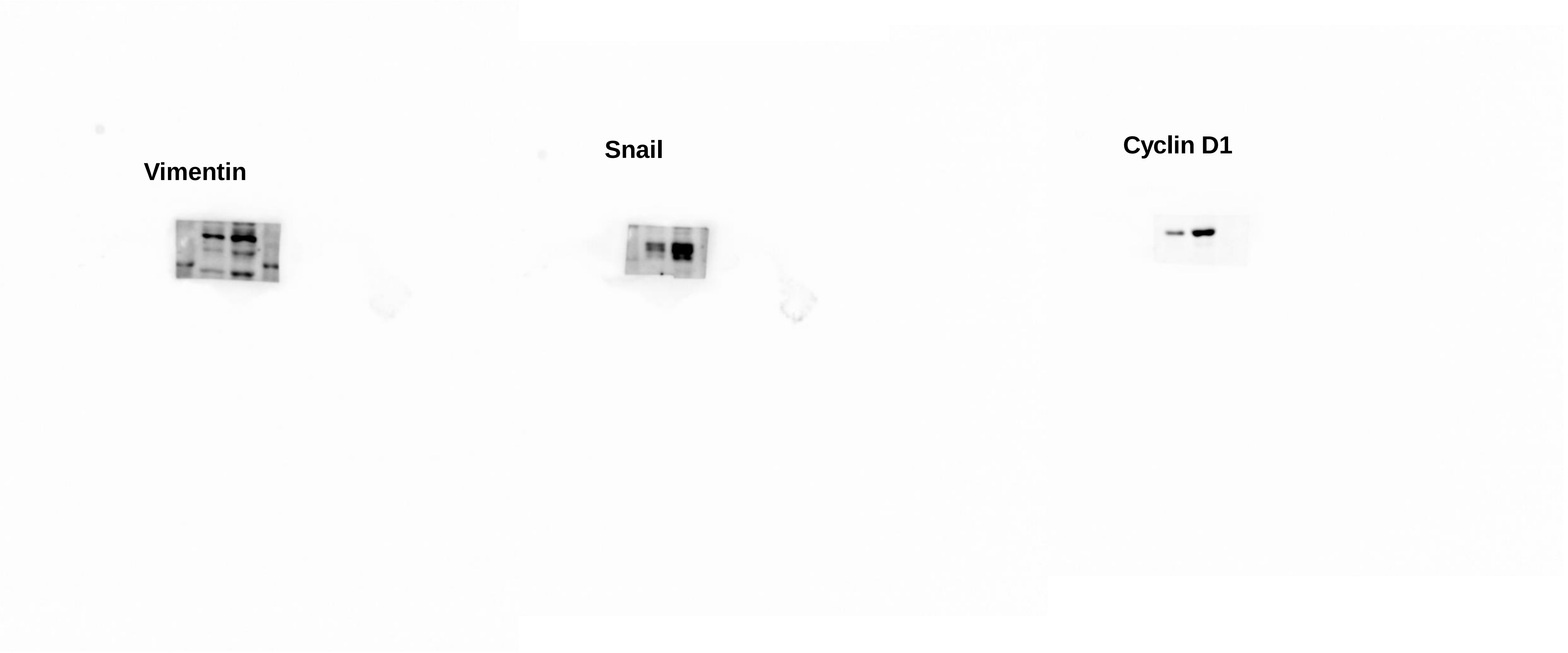

Figure S6A

β-catenin

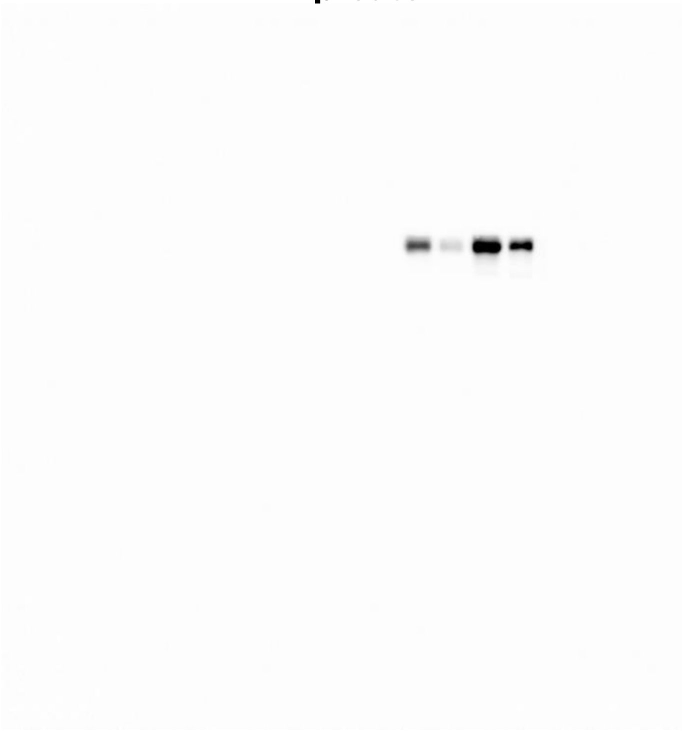

UM1 p-β-catenin

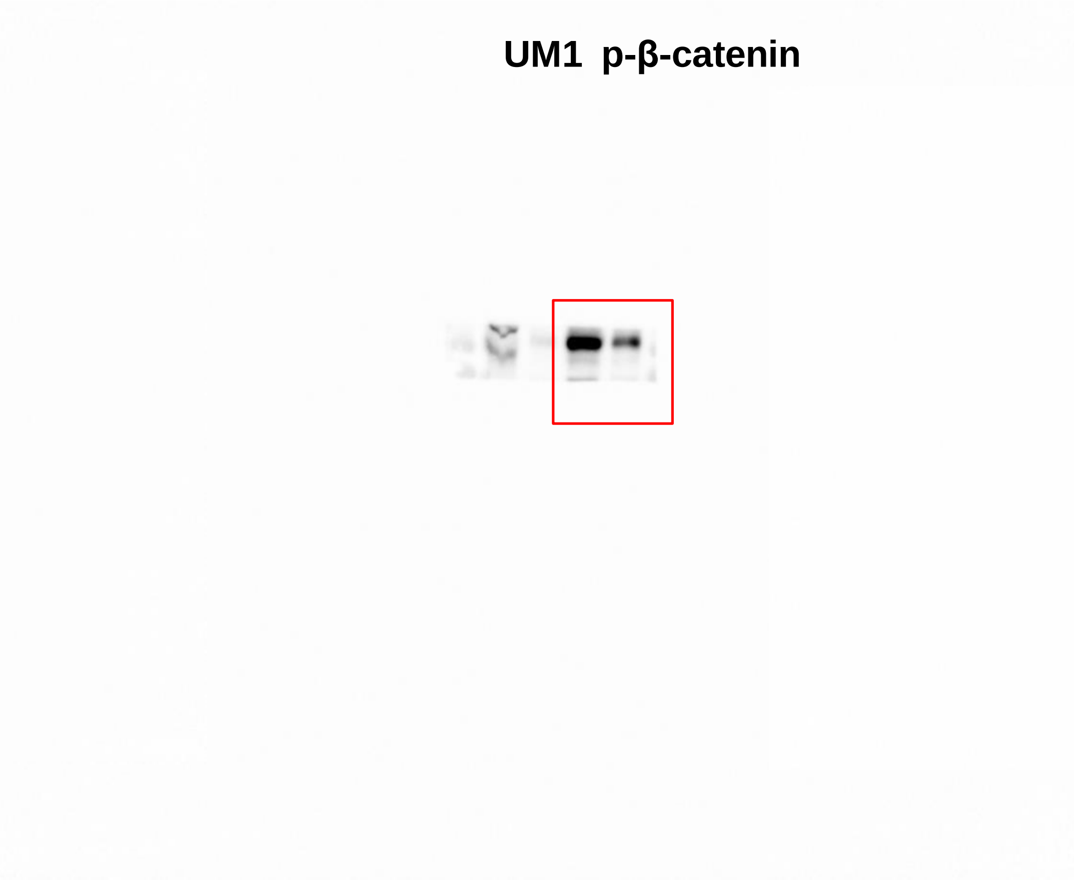

SCC15 p-β-catenin

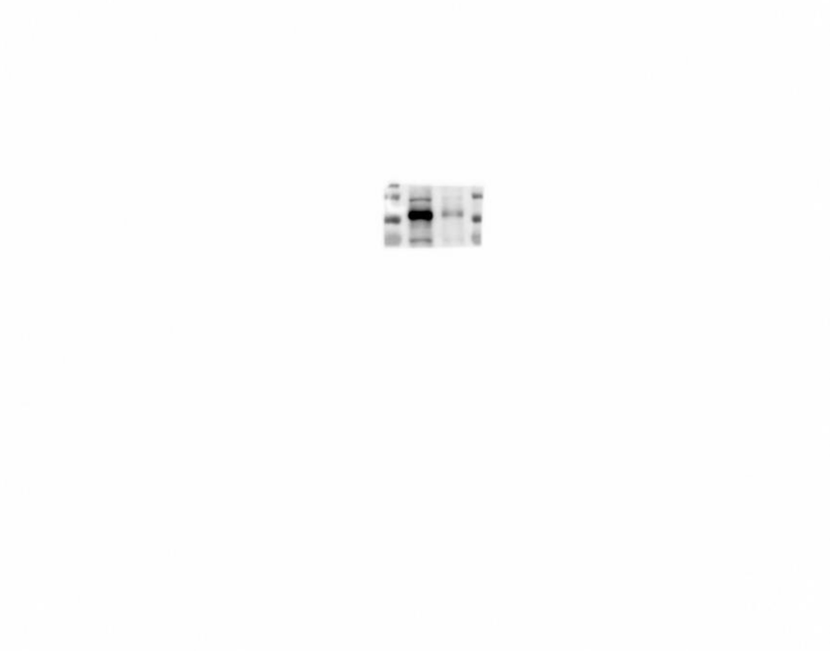

Figure S6A

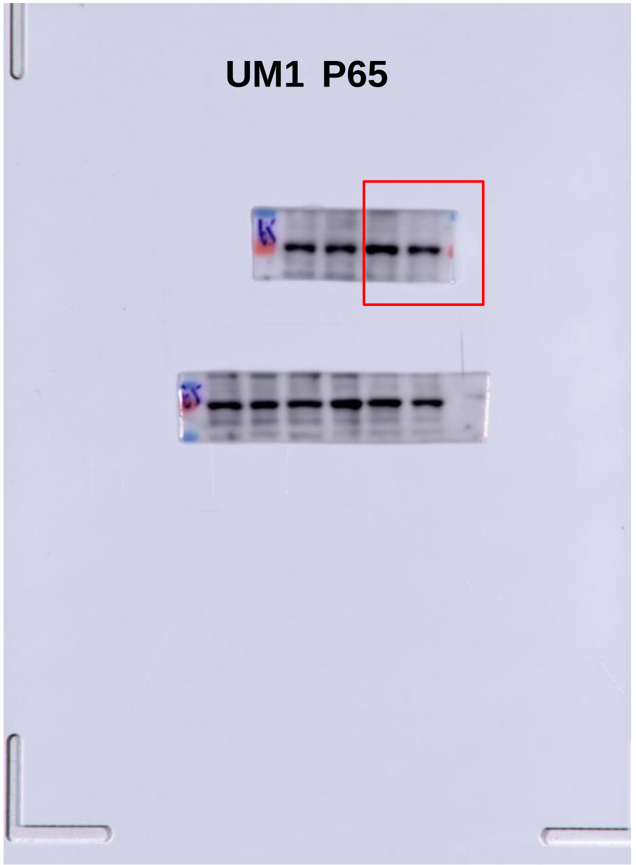

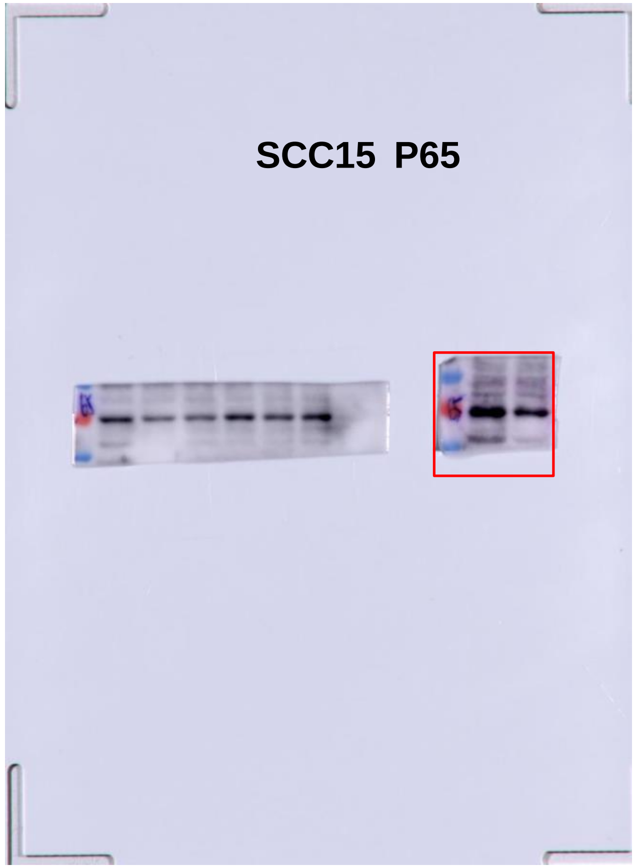

p-P65

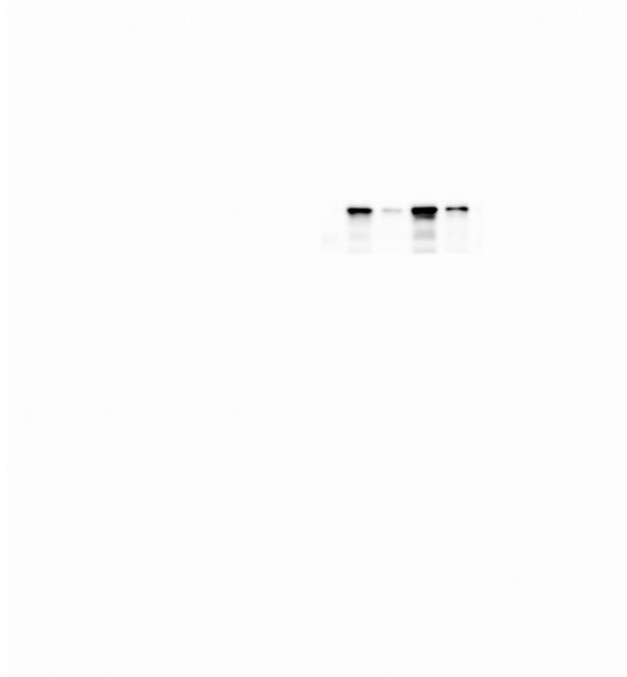

GAPDH

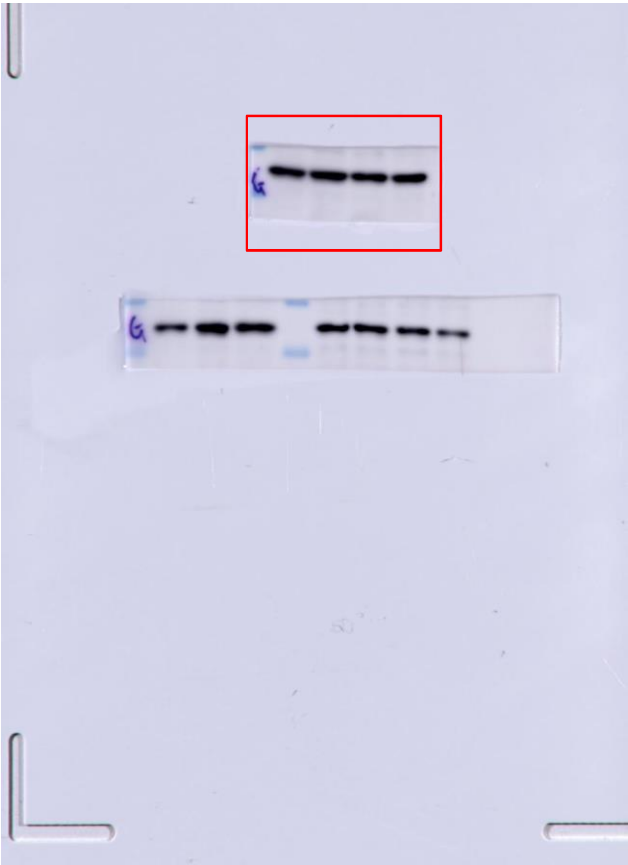

Figure S6B

cytoplasm

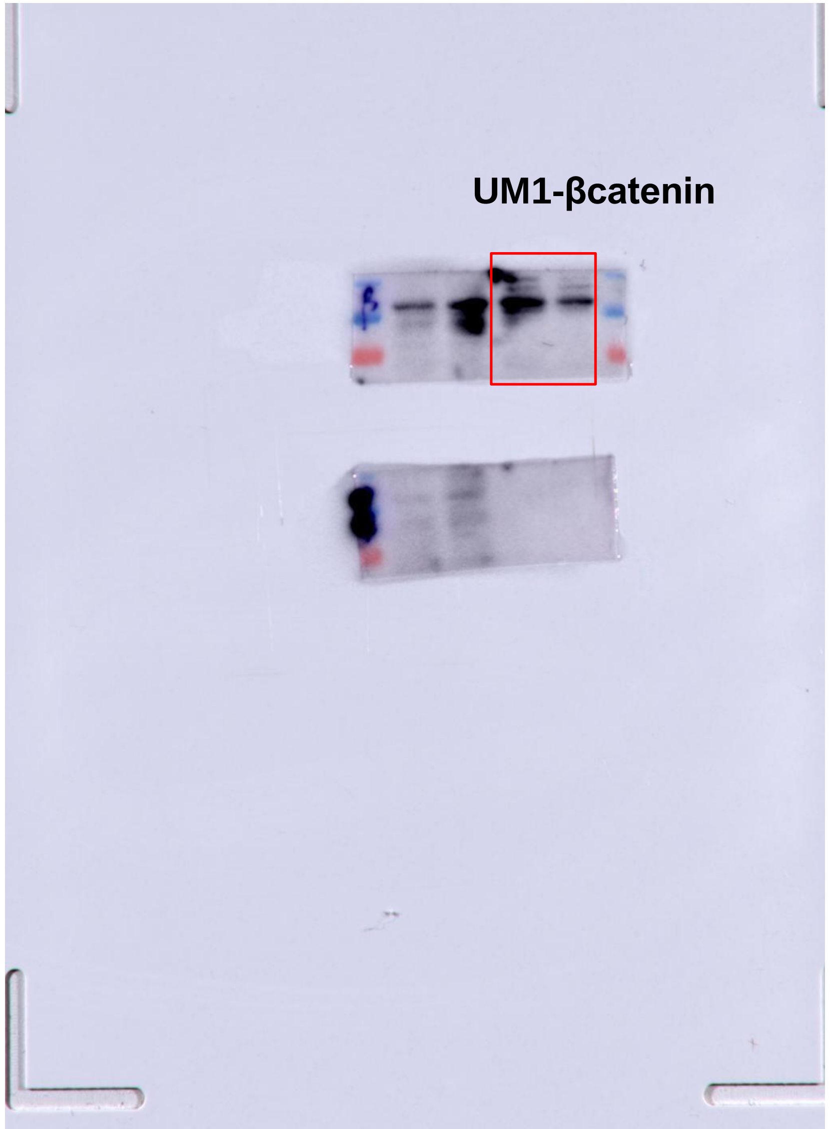

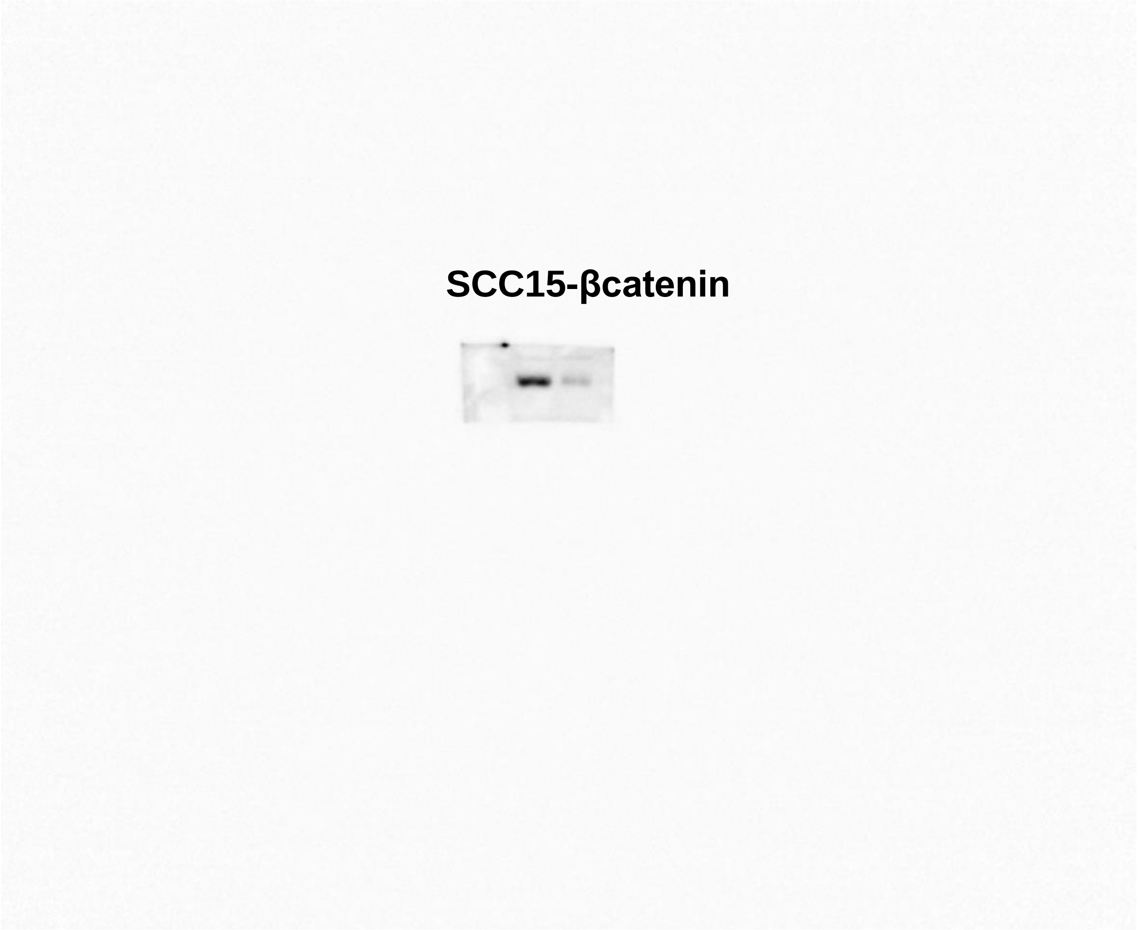

Figure S6B **cytoplasm**

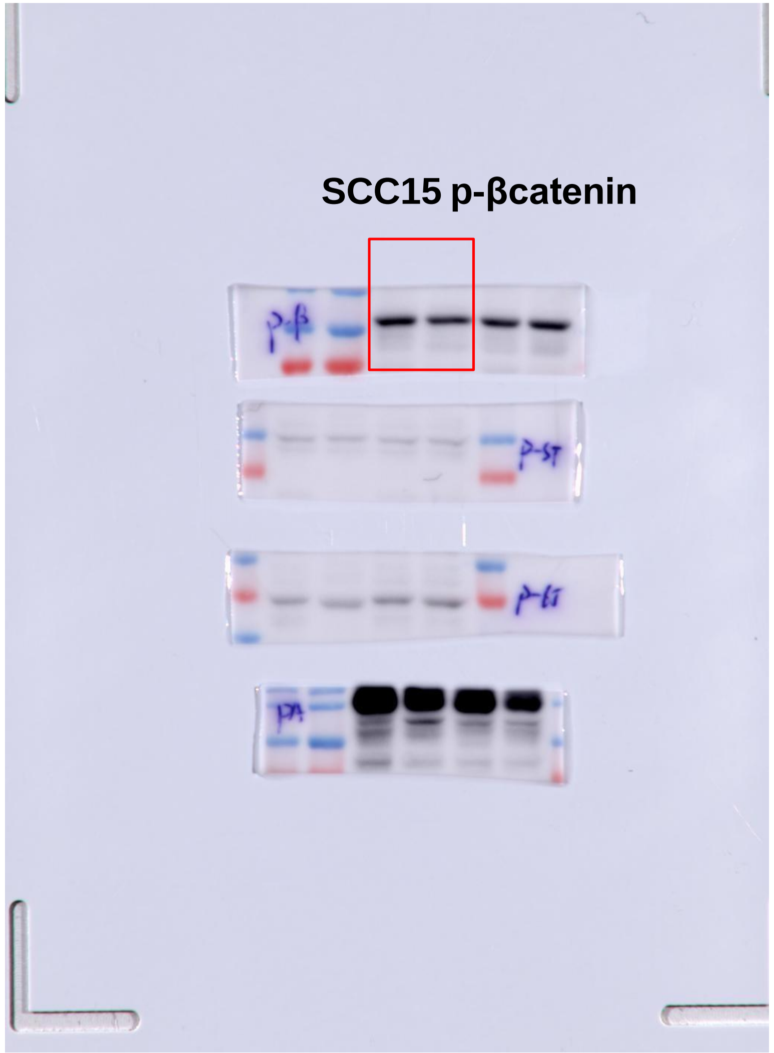

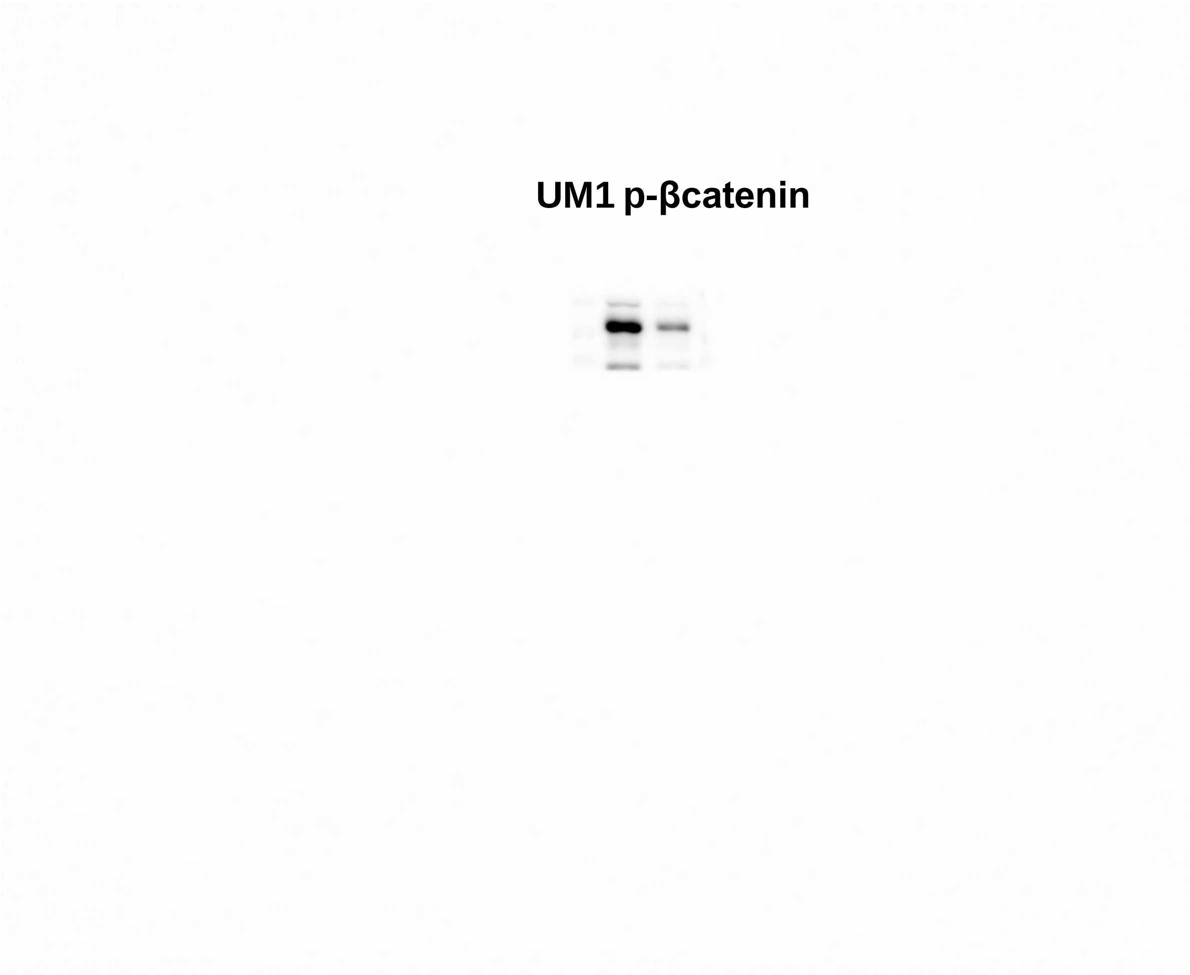

Figure S6B

cytoplasm

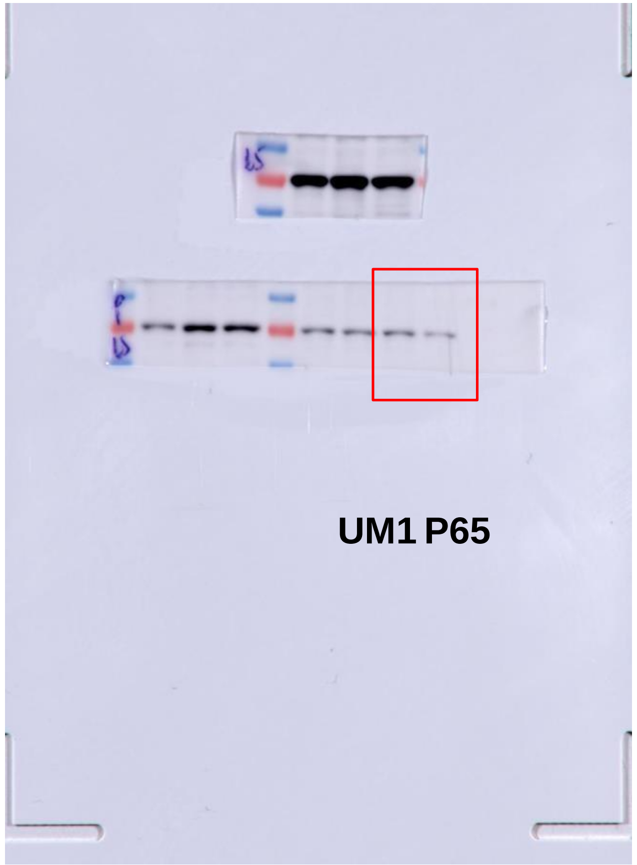

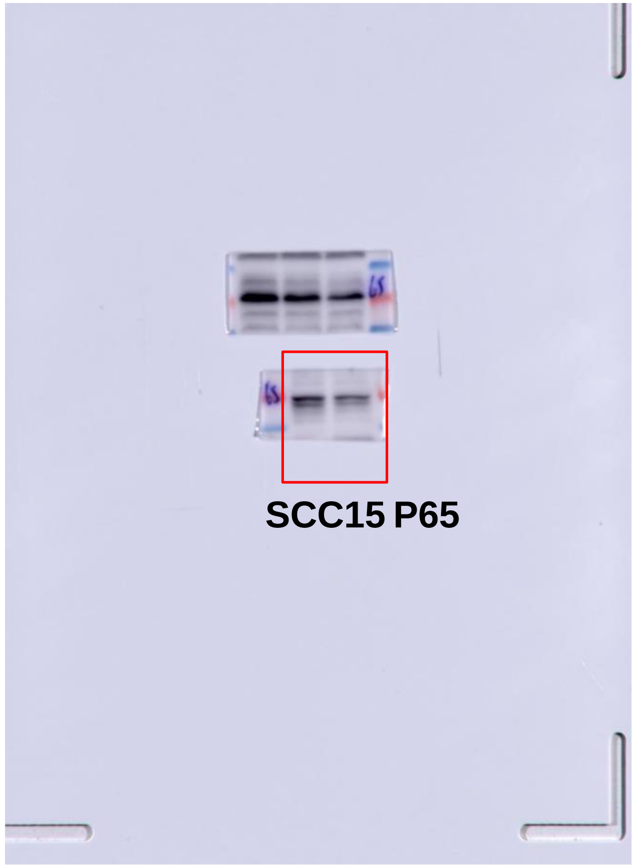

Figure S6B cytoplasm

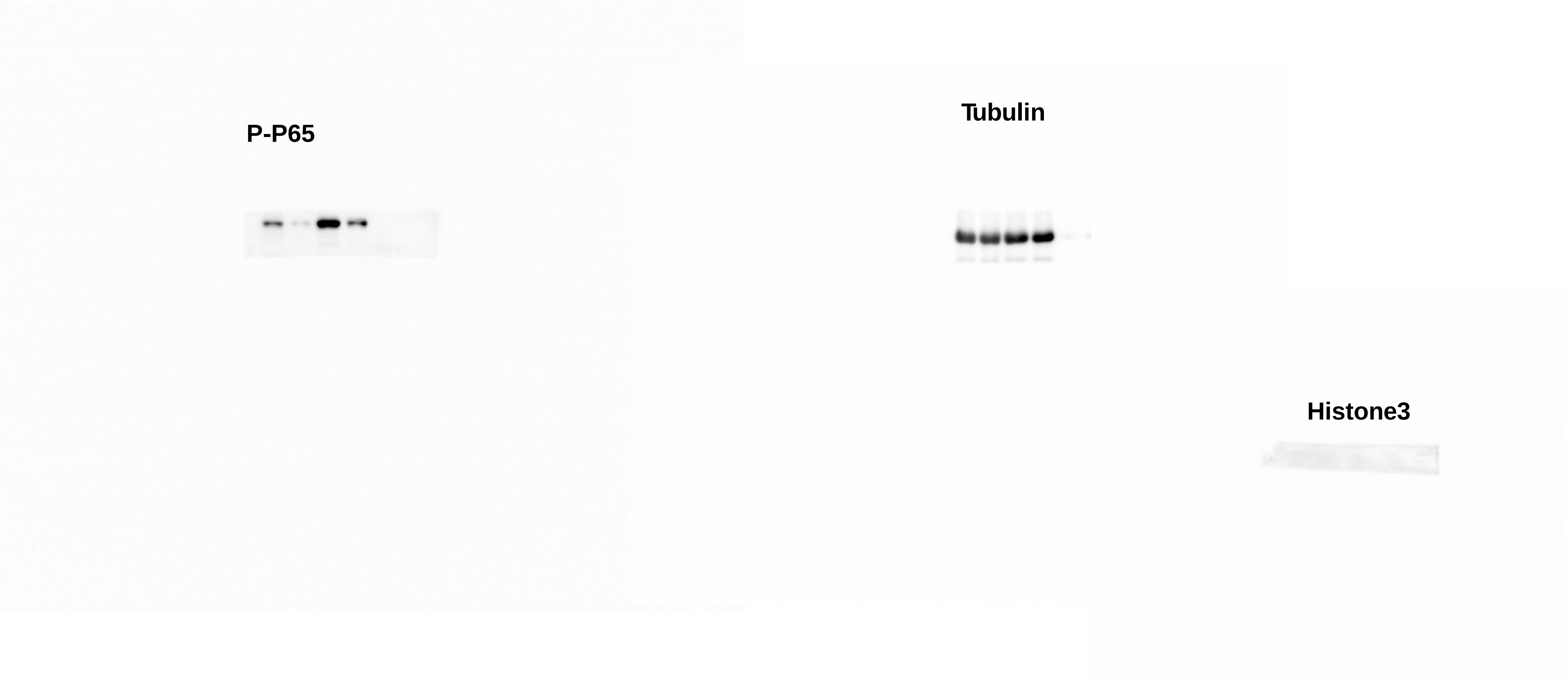

Figure S6B **nucleus**

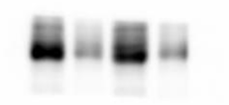

Figure S6B nucleus

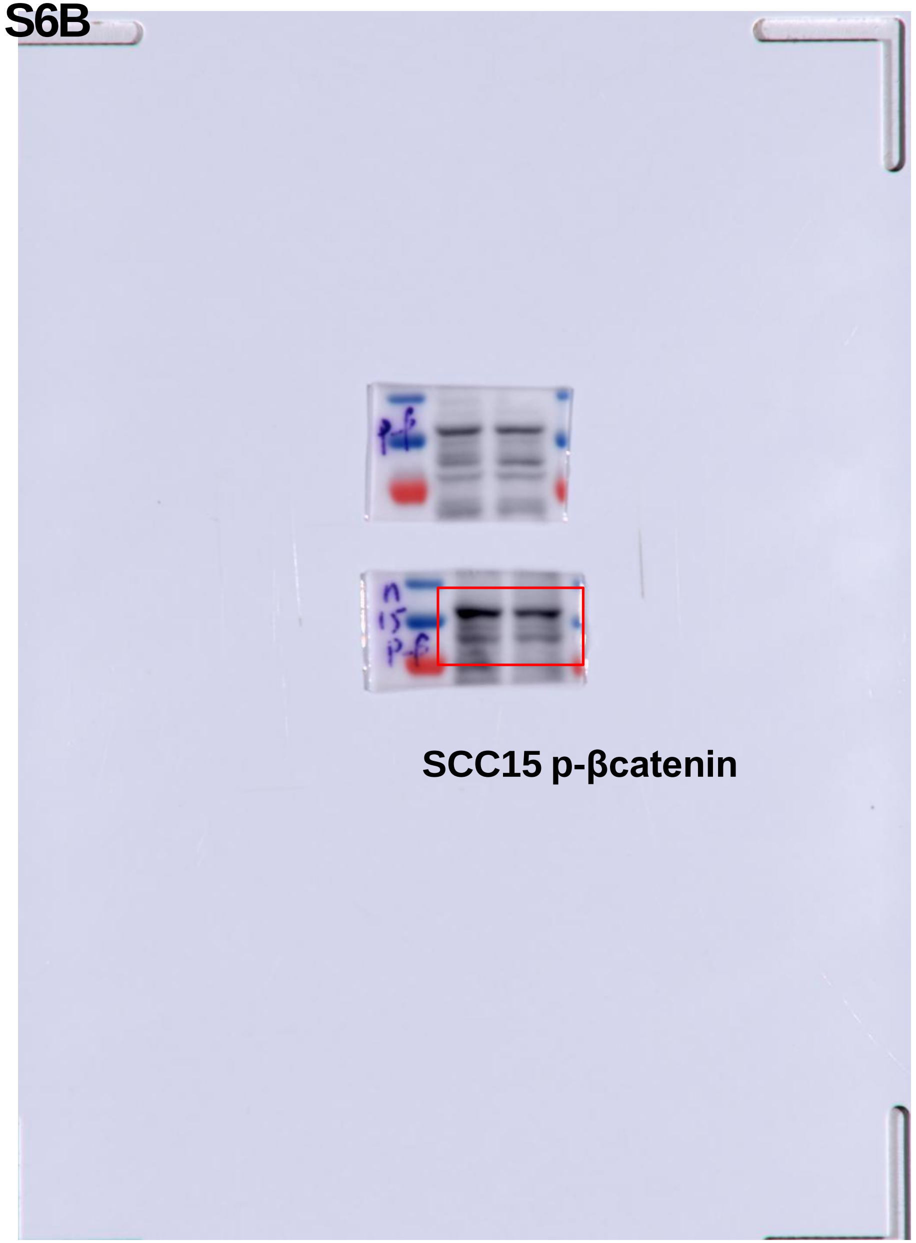

SCC15 p-βcatenin

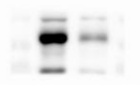

UM1 p-βcatenin

Figure S6B **nucleus**

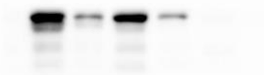

P65

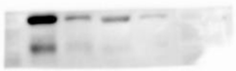

p-P65

Figure S6B **nucleus**

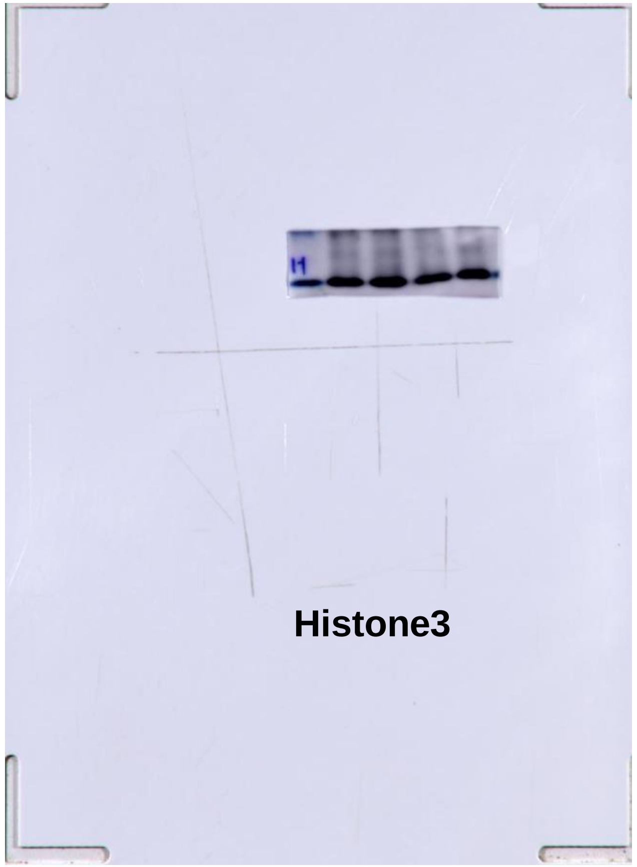

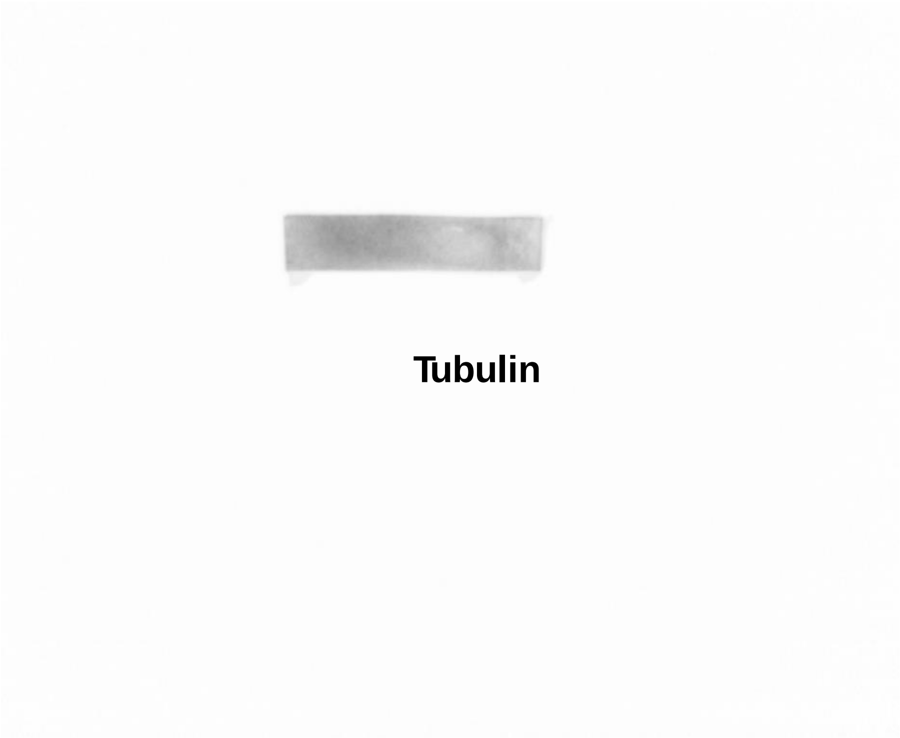

Supplement: Supplementary file 2 — Full and uncropped western blots [file 41419_2023_5667_MOESM2_ESM.pdf]
